# Evaluating methods for Lasso selective inference in biomedical research: a comparative simulation study

**DOI:** 10.1186/s12874-022-01681-y

**Published:** 2022-07-26

**Authors:** Michael Kammer, Daniela Dunkler, Stefan Michiels, Georg Heinze

**Affiliations:** 1 Section for Clinical Biometrics, Center for Medical Statistics, Informatics and Intelligent Systems, Medical University of Vienna, Vienna, Austria; 2 Division of Nephrology and Dialysis, Department for Internal Medicine III, Medical University of Vienna, Vienna, Austria; 3 Service de Biostatistique et d’Epidémiologie, Gustave Roussy, Oncostat U1018, INSERM, University Paris-Saclay, labeled Ligue Contre le Cancer, Villejuif, France

**Keywords:** Selective inference, Penalized regression, Linear model, Variable selection, Simulation study

## Abstract

**Background:**

Variable selection for regression models plays a key role in the analysis of biomedical data. However, inference after selection is not covered by classical statistical frequentist theory, which assumes a fixed set of covariates in the model. This leads to over-optimistic selection and replicability issues.

**Methods:**

We compared proposals for selective inference targeting the submodel parameters of the Lasso and its extension, the adaptive Lasso: sample splitting, selective inference conditional on the Lasso selection (SI), and universally valid post-selection inference (PoSI). We studied the properties of the proposed selective confidence intervals available via R software packages using a neutral simulation study inspired by real data commonly seen in biomedical studies. Furthermore, we present an exemplary application of these methods to a publicly available dataset to discuss their practical usability.

**Results:**

Frequentist properties of selective confidence intervals by the SI method were generally acceptable, but the claimed selective coverage levels were not attained in all scenarios, in particular with the adaptive Lasso. The actual coverage of the extremely conservative PoSI method exceeded the nominal levels, and this method also required the greatest computational effort. Sample splitting achieved acceptable actual selective coverage levels, but the method is inefficient and leads to less accurate point estimates.

The choice of inference method had a large impact on the resulting interval estimates, thereby necessitating that the user is acutely aware of the goal of inference in order to interpret and communicate the results.

**Conclusions:**

Despite violating nominal coverage levels in some scenarios, selective inference conditional on the Lasso selection is our recommended approach for most cases. If simplicity is strongly favoured over efficiency, then sample splitting is an alternative. If only few predictors undergo variable selection (i.e. up to 5) or the avoidance of false positive claims of significance is a concern, then the conservative approach of PoSI may be useful. For the adaptive Lasso, SI should be avoided and only PoSI and sample splitting are recommended. In summary, we find selective inference useful to assess the uncertainties in the importance of individual selected predictors for future applications.

**Supplementary Information:**

The online version contains supplementary material available at 10.1186/s12874-022-01681-y.

## Background

Statistical regression models are ubiquitous in the analysis of biomedical data, where advances in data collection and measurement technologies facilitate the consideration of more and more details of the underlying biological processes to describe, predict or explain an outcome variable [1]. However, to keep the results intelligible and communicable in clinical practice, sparse models including few covariates selected according to their relationship with the outcome are often preferred, and some form of statistical inference for the selected variables is desired [2]. Such post-selection inference cannot be performed using classical statistical approaches, as they rely on the assumption of a prespecified set of independent variables to be included in the model. This is no longer the case when the same data is used to, first, select a set of covariates, and second, to estimate their coefficients and conduct inference. Hence, the selected set of variables constitutes a random component of the model. This issue of selectively assessing hypotheses has affected regression modelling since variable selection techniques were introduced decades ago and resulting problems, such as a lack of replicability, have been discussed extensively in the literature [3–6].

Naturally, this also affects the very commonly used L1-penalized regression, i.e. the Lasso, and its extensions such as the adaptive Lasso, which became very popular after their introduction as they provide automated variable selection and scalability [7–9]. In recent years, numerous proposals for post-selection inference for the Lasso address different use-cases and methodological approaches [9, 10]: two-stage approaches (sample splitting and data carving [11–13]), bootstrap based [14–16], de-sparsified/de-biased Lasso [17–23], approaches controlling the expected number of false positive selections (e.g. Stability selection [24, 25], or knockoff filtering [26]), inference in the presence of many confounders [27, 28], and other conceptual approaches [29–31].

A particular strand of works adopted the general selective inference framework to provide post-selection inference for the Lasso [3, 11, 32–34]. The objective of this work was to review and empirically evaluate these recent approaches to selective inference for the Lasso in a neutral comparison study and investigate how well they extend to the adaptive Lasso. We also assessed the practical use of selective inference in a real-world data example. Our focus was on typical biomedical applications, in which the number of observations exceeds the number of variables, and where inference about the roles of single covariates in a model is desired. Such an investigation was recently encouraged to establish evidence-based state-of-the-art guidance [35].

## Methods

We adopt the notation of Berk et al. [3] and distinguish models by their 'active sets' of included variables. Thus, if the set of candidate predictors comprises $p$ variables, we write ${M}_{F} :=\left\{\mathrm{1,2}, \dots ,p\right\}$ for the full model using all predictors, and $M\subseteq {M}_{F}$ for any (fixed) submodel. We use the notation $\widehat{M}({\varvec{y}})$ (generally abbreviated as $\widehat{M}$) for the model chosen by variable selection, depending on the outcome ${\varvec{y}}=\left({y}_{i}\right)\in {\mathbb{R}}^{n}$. The vector of regression coefficients corresponding to a specific choice of predictors *M* is denoted as ${\beta }_{M }:= {\left({\beta }_{j,M}\right)}_{j\in M}\in {\mathbb{R}}^{\left|\mathrm{M}\right|}$ (if $M$ = ${M}_{F}$ we will omit the index). Similarly, we write ${\varvec{X}}=({x}_{ij})$ ${\in {\mathbb{R}}}^{{\varvec{n}}\times \boldsymbol{ }{\varvec{p}}}$ for the matrix of $n$ observations on the $p$ variables and ${{\varvec{X}}}_{M}$ for the matrix comprising only the variables in $M$.

### The Lasso

Classical linear regression models the conditional expectation of the outcome variable of interest ${\varvec{Y}}$ as ${\mathbb{E}}\left({\varvec{Y}}|{\varvec{X}}\right)={\beta }_{0}+X{\varvec{\beta}}$ for the fixed predictor matrix ${\varvec{X}}\in \boldsymbol{ }{\mathbb{R}}^{{\varvec{n}}\times \boldsymbol{ }{\varvec{p}}}$**,** with intercept ${\beta }_{0}\in {\mathbb{R}}$ and regression coefficients ${\varvec{\beta}}\in {\mathbb{R}}^{p}$. For inference, the components ${Y}_{i}$ of ${\varvec{Y}}$ are assumed to follow independent Gaussian distributions with expected values equal to the linear predictors ${\beta }_{0}+{\beta }_{1}{x}_{i1}+\dots +{\beta }_{p}{x}_{ip}$ and constant variance ${\sigma }^{2}$. Given the observed outcome vector ${\varvec{y}}\in {\mathbb{R}}^{n}$**,** the Lasso regression model is obtained by maximising the penalized likelihood

$${\mathrm{L}}_{P}\left({\varvec{\beta}}\right)=L\left({\varvec{\beta}}\right)-\lambda \sum_{j=1}^{p}|{\beta }_{j}|,$$

where $L\left({\varvec{\beta}}\right)=L({\varvec{\beta}}|{\varvec{X}},{\varvec{y}})$ is the likelihood function and $\lambda$ controls the impact of the penalization term defined by the ${L}_{1}$-norm of the regression coefficients [8]. This form of penalization induces variable selection by forcing some of the entries of the estimated  $\widehat{{\varvec{\beta}}}$ to exactly zero. The objective function of the Lasso is convex, such that efficient algorithms exist to compute estimates for a whole path of $\lambda$ values [36]. Thanks to its sparsity and computational accessibility, the Lasso was widely adopted in many fields of modern science. However, the Lasso suffers from several drawbacks, such as a high false positive selection rate and a bias towards zero for large coefficients [9, 37, 38].

The adaptive Lasso [7] addresses these issues by introducing weights in the penalty term ${\sum }_{j}{w}_{j}|{\beta }_{j}|$. These weights can be obtained, e.g., from reciprocal unpenalized regression coefficients as ${w}_{j}=1/{\left|{\widehat{\beta }}_{j}\right|}^{\gamma },$ using a second hyperparameter $\gamma$ to control the impact of the weights on the penalization. In contrast to the ordinary lasso, the adaptive Lasso offers consistent variable selection under conditions that can be considered realistic with large sample sizes [7, 39]. The adaptive Lasso can be easily implemented in any software that is able to fit the ordinary Lasso by re-scaling the input data: weighting the contributions of individual coefficients $\left|{\beta }_{j}\right|$ to the penalty term by ${w}_{j}>0$ is equivalent to scaling the corresponding column in ${\varvec{X}}$ by $1/{w}_{j}$.

### Selective inference for the Lasso

Selective inference is a general paradigm to address issues arising when statistical hypotheses are not specified before data collection, but defined during the process of data analysis. The prototypical use-case presented here is the use of variable selection procedures [5]. In classical statistical inference, a $1-\alpha$ confidence interval (CI) $C{I}_{j}$ for a regression coefficient  ${\beta }_{j}$ is a contiguous set of numbers computed from the data such that the probability ${\mathbb{P}}\left[{\beta }_{j}\in C{I}_{j}\right]$ to cover the true parameter ${\beta }_{j}$ is $1-\alpha$, where $1-\alpha$ is a pre-specified confidence level. In analogy, selective CIs can be defined by model-dependent coverage probabilities. These apply when the researcher conducts variable selection to obtain a model $M$, and subsequently is interested in inference for the variables in $M$. An important quantity in the definition of selective coverage is the target parameter of inference in the population. The methods discussed in the following introduce the submodel population regression coefficients ${{\varvec{\beta}}}_{M}$ specific to a given model $M$ as inference targets. In the case of linear regression these are defined by ${{\varvec{\beta}}}_{M}:={\left({{\varvec{X}}}_{M}^{T}{{\varvec{X}}}_{M}\right)}^{-1}{{\varvec{X}}}_{M}^{T}{\mathbb{E}}\left({\varvec{Y}}\right)$ [3]. The submodel target is a linear functional of the true full model population parameters ${\varvec{\beta}}$, since ${{\varvec{\beta}}}_{M}={\left({{\varvec{X}}}_{M}^{T}{{\varvec{X}}}_{M}\right)}^{-1}{{\varvec{X}}}_{M}^{T}{\varvec{X}}{\varvec{\beta}}$**.** Note that ${{\varvec{\beta}}}_{M}$ is neither intended nor required to recover the corresponding true full model parameters in ${\varvec{\beta}}$**,** and its components will differ from the full model target ${\varvec{\beta}}$ unless there is no correlation between variables, which is unrealistic with observational data**.** Instead, the submodel target can be interpreted as the coefficients of a linear approximation to the full model using only a subset of variables. A brief discussion of alternative targets for inference is provided in Supplementary Figure S1 and in the original publication by Berk et al. [3].

#### Sample splitting (Split)

Sample splitting is an intuitive approach to selective inference agnostic to the model selection procedure, introduced already in 1975 [40]. It consists of partitioning the dataset into two parts. First, a set of active variables $M$ is derived from one part of the dataset. Inference is conducted using the other part of the dataset, in which the set of active variables can be considered fixed, conditional on the data used in the first step. Thus, classical statistical theory yields selective inference for $M$. This approach controls the submodel coverage at the nominal significance level $\alpha$ such that ${\mathbb{P}}\left[{\beta }_{j,M}\in C{I}_{j,M}|\widehat{M}=M\right]\ge 1-\alpha$, $j\in M$. Sample splitting is easily implemented in any statistical software. The two parts of the dataset can be of unequal sizes, related to a trade-off between selection and inference accuracy. Simulations suggest that a simple 50-50% split offers a good compromise [11, 30].

#### Exact post-selection inference for the Lasso (SI)

The procedure proposed by Lee et al. [33] constructs selective CIs that guarantee coverage at the nominal significance level $\alpha$, conditional on the specific model $M$ that was selected by the Lasso such that $P\left[{\beta }_{j,M}\in C{I}_{j,M}| \widehat{M}=M\right]\ge 1-\alpha$, $j\in M$. The authors show that the selection event for $M$ corresponds to a polyhedral region in the space ${\mathbb{R}}^{p}$ of regression coefficients, and are thereby able to analytically derive the sampling distribution conditional on $M$ required to compute CIs. The approach assumes an estimate of the outcome variance ${\sigma }^{2}$ to provide valid inference, in practice estimated using the squared residual error from the full unpenalized model as ${\widehat{\sigma }}^{2}=\frac{1}{n-p-1}\sum_{i=1}^{n}{\left({y}_{i}-{\widehat{y}}_{i,F}\right)}^{2}$. Notably, this method was derived for the special case of the Lasso with a fixed parameter $\lambda$. In practice, this is not a realistic usage scenario as the penalization strength is generally tuned. Computer intensive extensions to incorporate tuning of $\lambda$ have been developed, but are not yet available as a software package [30, 41].

#### Universally valid post-selection inference (PoSI)

The approach by Berk et al. [3] was developed to provide valid CIs irrespective of model selection strategy. For a given significance level $\alpha$, the authors propose to control the family wise error rate $\forall \widehat{M}\subseteq {M}_{F}:{\mathbb{P}}\left[\forall j\in \widehat{M}:{\beta }_{j,\widehat{M}}\notin C{I}_{j,\widehat{M}}\right]\le \alpha ,$ casting selective inference as a multiple testing problem. For any specific selected submodel $M$ and its estimated coefficients ${\widehat{{\varvec{\beta}}}}_{M}$, symmetric confidence intervals are formed as $C{I}_{j,M}=\left[{\widehat{\beta }}_{j, M} \pm K \widehat{\sigma }\left({\widehat{\beta }}_{j, M}\right)\right]$, where $\widehat{\sigma }\left({\widehat{\beta }}_{j, M}\right)$ denotes the estimated standard error of the $j$-th entry of the coefficient vector ${\widehat{{\varvec{\beta}}}}_{M}$. The “PoSI” multiplier $K$ is computed to account for the selection of $M$ from the space of all submodels of ${M}_{F}$. It depends on the correlation structure of the dataset**,** desired coverage and an independent estimate of the outcome variance ${\sigma }^{2}$. The latter may be based on the squared residual error of the full model, assuming it is correctly specified. Other possible ways of estimation such as using another independent dataset to estimate the variance were outlined in the original publication. As there is no general closed-form expression for $K$, it is approximated by Monte-Carlo simulation. This method providing simultaneous error control is expected to be a very conservative procedure, but recent extensions [4] or restricting the search space of submodels may improve efficiency of the method.

### Simulation design

Our simulation study is reported following the ADEMP-structure by Morris et al. [42]. A brief simulation profile as well as in-depth details can be found in the Supplementary Material Sects. 2 and 3.

#### Aim

The aim of this simulation study was to evaluate recent proposals for selective inference in the context of Lasso regression regarding their frequentist properties in practical usage scenarios.

#### Data generation

We generated data using a generic simulation procedure: first, we sampled a matrix ${\varvec{Z}}$ from a multivariate normal distribution with pre-specified correlation structure. The resulting values were then transformed (denoted by ${\varvec{T}}$) to obtain different predictor distributions. Given the final data matrix ${\varvec{X}}={\varvec{T}}({\varvec{Z}})$ of pre-specified sample size, we computed the true outcome ${\varvec{y}}={\varvec{X}}{\varvec{\beta}}+{\varvec{\epsilon}}$ using a pre-specified coefficient vector ${\varvec{\beta}}$. The signal-to-noise ratio (target coefficient of determination R^2^) was controlled via the variance of ${\varvec{\epsilon}}$ drawn from a normal distribution with mean zero. Validation data was obtained by using the same realisation of the data matrix ${\varvec{X}}$ and drawing another error vector ${\varvec{\epsilon}}\boldsymbol{^{\prime}}$ to obtain a new outcome vector $\boldsymbol y\boldsymbol'$. 

Using this generic simulation procedure, we created two different setups: a simple ‘toy setup’ with multivariate normal data (${\varvec{T}}$ is the identity function) using various correlation structures, and a ‘realistic setup’ with more realistic distributions and dependencies (Table 1). For the latter, ${\varvec{T}}$ comprised affine and exponential functions, as well as thresholding to yield continuous and binary variables following Binder et al. [43].

**Table 1:** Simulation setup

		**Toy setup**	**Realistic setup**
**Fixed design parameters**	Motivation	Simplicity, insight	Realistic data
	Number of variables	4	17
	Type of variables	Continuous	Continuous, binary
	Distribution of variables	Gaussian	Mixed (Supplementary Table S2)
**Varying design parameters**	Correlation structures ${\varvec{\Sigma}}$	7 blocked correlation matrices with no or strong correlation (Supplementary Material Sect. 3.2)	Fixed, mimicking real study (Supplementary Material Sect. 3.3)
	Coefficient structures ${\varvec{\beta}}$	10 (Supplementary Table S1)	13 (Supplementary Table S3)
	True target R^2^ (noise ${\varvec{\epsilon}}$)	0.2, 0.5, 0.8	0.2, 0.5, 0.8
	Observations per variable	5, 10, 50	5, 10, 50
**Simulation parameters**	Number of scenarios	630	117
	Iterations per scenario	900	900

See the description of the simulation setups in the Supplementary Material for more details on the correlation and coefficient structures. Varying design parameters are the parameters that were varied in the full factorial design in both setups

#### Estimands and other targets

The primary estimands of interest in our simulation were the selective CIs, i.e. the lower and upper confidence limits, for the methods under evaluation. The confidence level was fixed at 90%, which is also the default level in the main software package used. Other targets of interest were the selective null hypotheses associated with the CIs, the selected submodels, and their predictions.

#### Methods for simulation study

Methods studied in our work consisted of a variable selection step, and a subsequent inference step (Table 2). Variable selection was conducted using the Lasso (Lasso) and the adaptive Lasso (ALasso). We used the reciprocals of the absolute values of the coefficient estimates from the unpenalized full model as penalization weights in the adaptive Lasso. The penalization parameters were determined via tenfold cross-validation (CV, estimated through the parameter that yielded the smallest CV prediction error) or, for the SI method only, via the method by Negahban et al. (Neg, [44]), which differ in whether or not the observed outcome is used in the estimation procedure (Supplementary Material Sect. 3.4).

**Table 2:** Overview of methods investigated in this study

Method	Variable selection	Tuning	Inference
Full	None	None	Wald CI
Oracle	None	None	Wald CI
Lasso-CV-Split	Lasso	tenfold CV	Split-sample
Lasso-CV-PoSI	Lasso	tenfold CV	Universally valid post-selection inference [3]
Lasso-CV-SI	Lasso	tenfold CV	Exact post-selection inference [33]
Lasso-Neg-SI	Lasso	Fixed penalization parameter [44]	Exact post-selection inference [33]
ALasso-CV-Split	Adaptive Lasso	tenfold CV	Split-sample
ALasso-CV-PoSI	Adaptive Lasso	tenfold CV	Universally valid post-selection inference [3]
ALasso-CV-SI	Adaptive Lasso	tenfold CV	Exact post-selection inference [33]
ALasso-Neg-SI	Adaptive Lasso	Fixed penalization parameter [44]	Exact post-selection inference [33]

In the inference step, we evaluated the Split, SI and PoSI methods for selective inference.

For comparison, we included two methods without data-driven variable selection: the full model (Full) on all variables and the oracle model (Oracle), which included only the variables used for generating the data. The oracle model served as a benchmark and was generally expected to perform best among all methods, since it "knew" the true data generating mechanism. Of course, such information is not available during a real analysis and therefore was inaccessible to all other methods.

#### Performance measures

We evaluated three primary performance measures for selective CIs, marginalizing over all selected models and variables (Table 3):

- Selective (actual) coverage probability was estimated as the proportion of simulation iterations in which the CI covered the submodel target parameter; methods with coverage probability closer to the nominal confidence level were considered better.
- Selective power for a variable with submodel target parameter unequal to zero was estimated by the proportion of simulation iterations in which its corresponding CI excluded zero; methods with higher power were considered better.
- Selective type 1 error probability for a variable with submodel target parameter equal to zero was estimated as the proportion of simulation iterations in which the CI excluded zero; methods with error probability closer to one minus the nominal confidence level were considered better.

**Table 3:** Primary performance measures investigated in this study

Measure	Definition	Approximation by simulation
Coverage	${\mathbb{P}}\left[{\beta }_{.,\widehat{M}}\in C{I}_{.,\widehat{M}}\right]$	$\frac{{\sum }_{j\in {M}_{F}}{\sum }_{M\subseteq {M}_{F}}{\sum }_{s\in S}{\mathbb{I}}\left[{\widehat{M}}_{s}=M\wedge {\beta }_{j,M}\in C{I}_{j,M}\right]}{{\sum }_{j\in {M}_{F}}{\sum }_{s\in S}{\mathbb{I}}\left[j\in {\widehat{M}}_{s}\right]}$
Power	${\mathbb{P}}\left[{\beta }_{.,\widehat{M}}\in C{I}_{.,\widehat{M}}|{\beta }_{.,\widehat{M}}\ne 0\right]$	$\frac{\sum_{j\in {M}_{F}}{\sum }_{M\subseteq {M}_{F}}{\sum }_{s\in S}{\mathbb{I}}\left[{\widehat{M}}_{s}=M\wedge 0\notin C{I}_{j,M}\right]}{{\sum }_{j\in {M}_{F}}{\sum }_{s\in S}{\mathbb{I}}\left[j\in {\widehat{M}}_{s}\wedge {\beta }_{j,{\widehat{M}}_{s}}\ne 0\right]}$
Type 1 error	${\mathbb{P}}\left[{\beta }_{.,\widehat{M}}\in C{I}_{.,\widehat{M}}|{\beta }_{.,\widehat{M}}=0\right]$	$\frac{\sum_{j\in {M}_{F}}{\sum }_{M\subseteq {M}_{F}}{\sum }_{s\in S}{\mathbb{I}}\left[{\widehat{M}}_{s}=M\wedge 0\notin C{I}_{j,M}\right]}{{\sum }_{j\in {M}_{F}}{\sum }_{s\in S}{\mathbb{I}}\left[j\in {\widehat{M}}_{s}\wedge {\beta }_{j,{\widehat{M}}_{s}}=0\right]}$

We denote the set of all iterations of a simulation scenario by $S=\{1,\dots ,{n}_{sim}\}$. The full model using all predictors is written as ${M}_{F}=\{1,\dots ,p\}$, the selected model in a specific iteration $s$ is written as ${\widehat{M}}_{s}$. By the use of ${\mathbb{I}}[.]$ we denote the indicator function for the event specified between square brackets. Note that for methods without variable selection, the estimands reduce to the usual definitions of frequentist properties. More details on the derivation of the approximation in the simulation are given in the Supplementary Material Sect. 3.1

In the computations of these performance measures the submodel targets ${{\varvec{\beta}}}_{\widehat{M}}$ depend on the selected model $\widehat{M}$ and can vary across the iterations of the simulation. They were computed for each iteration using the true covariance matrix and the pre-specified population values of the full vector of regression coefficients ${\varvec{\beta}}$**.** In particular, ${{\varvec{\beta}}}_{\widehat{M}}={{\varvec{\Sigma}}}_{\widehat{M}}{{\varvec{\Sigma}}}_{\widehat{M},{M}_{F}}{\varvec{\beta}},$ where ${{\varvec{\Sigma}}}_{\widehat{M}}$ is the $q\times q$ submatrix of the true covariance matrix ${\varvec{\Sigma}}$ which contains only the variables in $\widehat{M}$, and ${{\varvec{\Sigma}}}_{\widehat{M},{M}_{F}}$ is the $q\times p$ submatrix which contains all the covariances of the variables in $\widehat{M}$ and the whole set of candidate predictors ${M}_{F}$.

We also report median and interquartile ranges of widths of selective CIs as additional performances measures. Since the SI method has been shown to lead to highly variable CIs of extreme or even infinite width in some cases [33, 45], we report the relative frequency with which this occurred. To address the remaining study targets, we report simulation estimates for the probability of true model selection and variable selection probabilities, for which methods closer to the oracle model were considered better. Relative prediction performance of the models was measured by the achieved R^2^ on validation data, where higher was considered better.

### Extensions of the main simulation design

Besides the simulation designs outlined so far, we have also assessed several more specific research questions using smaller setups:

- To study the properties of selective CIs for different confidence levels, we assessed the selective coverage for 95% selective CIs in a setup based on the toy setup, but using only 300 iterations per scenario.
- We focus on the use of CV to tune penalization parameters due to its widespread use. Another commonly used method is the closely related Akaike information criterion (AIC). Both tune penalization strength by an estimate of the out-of-sample prediction error. Alternatively, the Bayesian information criterion (BIC) may also be used. We assessed the properties of selective CIs when the Lasso is tuned with AIC and BIC instead of CV in a smaller simulation study based on the toy setup, but using the SI method only. The other inference methods (Split and PoSI) are unaffected by the change in tuning strategy.
- We assessed whether the results transfer to logistic regression in a smaller simulation study based on the realistic setup, but in which the events per variable (5, 10, 50) and outcome prevalence (0.1 or 0.5) were varied. Only 4 methods (Oracle, Full, Lasso-CV-Split, Lasso-CV-SI) were evaluated in this smaller setup, as the PoSI method is not directly available in this setting so far.

### Software and implementation details

All analyses were implemented in the R statistical software, version 3.5.1 [46]. We used the packages *glmnet* [47] (version 2.0–18)*,* for implementing Lasso and ALasso, *selectiveInference* [48] (version 1.2.4) for the SI and *PoSI* [49] (version 1.0) for the PoSI method. Data simulation and visualisation was facilitated by the *simdata* [50] and *looplot* [51] packages. Generally, we left most options for the selectiveInference and PoSI packages at their sensible default values, but adapted several arguments for computational feasibility, see Supplementary Material Sect. 3.5 for details. In line with the literature, we derived estimates of the outcome variances required for the SI and PoSI methods from the residuals of the full model fitted on the training data set. All computations were done on standardized covariate data.

## Results

### Selective inference

#### Primary estimands

**Fig. 1:**
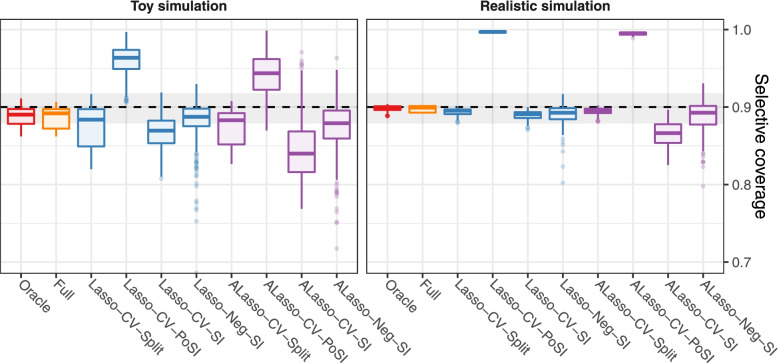
Simulation study: selective coverage from both simulation setups of selective 90% CIs for the submodel inference target. For each scenario, the actual selective coverage rate was estimated by simulation, and over all scenarios, the values were summarised by boxplots. See Supplementary Figure S4 for stratified results. The nominal confidence level of 0.9 used in the construction of the CIs is depicted as dashed line. Colors indicate the type of variable selection. Monte Carlo error is indicated by grey areas describing binomial 95% CIs expected at the nominal confidence level with 900 iterations

**Fig. 2:**
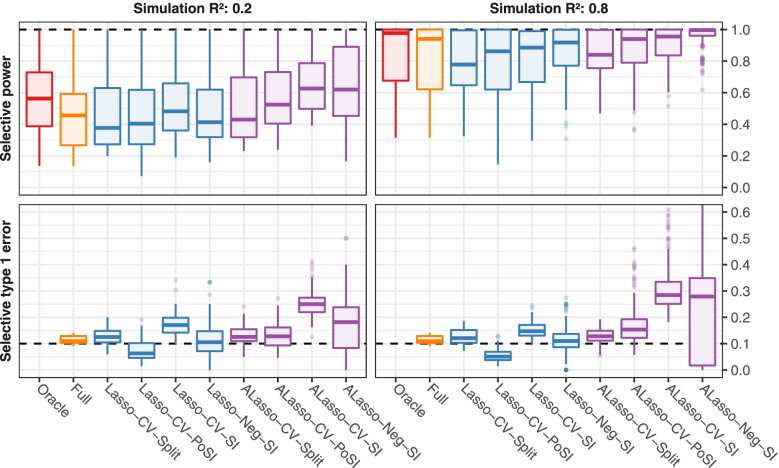
Toy simulation study: selective power and selective type 1 error of selective 90% confidence intervals. For each scenario, power or type I error was estimated by simulation, and over all scenarios with specified target simulation R^2^, the values were summarised by boxplots. The target values are depicted as dashed lines (1 for power, 0.1 for type 1 error). Colors indicate the type of variable selection. Results from the realistic setup are comparable to the ones shown here (Supplementary Figure S5)

Summary results for selective coverage in both simulation setups are shown in Fig. 1, with a more in-depth view provided in Supplementary Figure S4. Selective power and type 1 error are depicted in Fig. 2 and Supplementary Figure S5 for the toy and realistic setups, respectively. Generally, the realistic setup showed lower variability since it was based on a fixed correlation structure, in contrast to the toy setup. Selective coverage and type 1 error remained stable across the different signal-to-noise ratios and were similar between both simulation setups, while selective power increased with higher signal-to-noise ratio. Lasso-CV-SI led to coverage rates slightly lower than nominal (in median 0.03 below target for the toy setup, less than 0.01 for the realistic setup). This was partly a result of computational issues: in the realistic setup a relevant proportion of simulation iterations (26%) led to highly variable CIs by the SI method (see the section on 20 below). Removing such runs from the analyses shifted results towards claimed levels: median selective coverage over all scenarios in the realistic setup increased to 0.93, median selective type 1 error decreased from 0.12 to 0.06. As expected, Lasso-CV-PoSI and ALasso-CV-PoSI were conservative, especially in the realistic setup with a larger number of variables, and yielded coverage rates above 0.9 with corresponding selective type 1 error rates clearly below 0.05. In contrast to the Lasso, ALasso-CV-SI consistently led to noticeably lower-than-nominal selective coverage (in median 0.06 below target in the toy setup). While the selective power was higher than for the Lasso, the selective type 1 error also increased drastically. Lasso-CV-Split and ALasso-CV-Split gave similar results, as sample splitting is agnostic of the model selection procedure.

Additional results for selective coverage conditional on a specific variable being selected are shown in Supplementary Figure S6. The results indicate that coverage generally decreases with decreasing variable selection frequency for the PoSI and SI methods and is particularly problematic with the adaptive Lasso.

#### Widths of confidence intervals

**Fig. 3:**
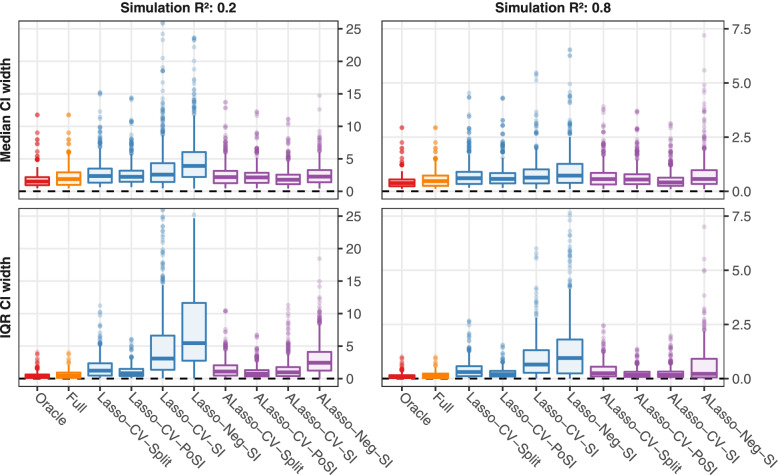
Toy simulation study: median and interquartile range (IQR) of the widths of selective 90% CIs. CIs were standardized. For each scenario the median and IQR of CI widths were computed, and over all variables and scenarios with specified target simulation R^2^, the values were summarised by boxplots. Dashed lines mark a width of zero. Colors indicate the type of variable selection. Supplementary Figure S7 shows the same results stratified by true predictors and noise variables. Results for the realistic setup are comparable (Supplementary Figure S8)

Figure 3 and Supplementary Figures S7 and S8 depict summaries of the results regarding the width of the selective CIs. Widths of CIs were standardized and corresponded to unit standard deviation of a predictor. The shortest intervals were obtained by the comparator methods without data-driven selection (i.e. the Oracle followed by Full). The intervals obtained by the SI method (Lasso-CV-SI, ALasso-CV-SI, Lasso-Neg-SI, ALasso-Neg-SI) could be extremely wide, usually when a weak or noise variable was selected into the active set. However, these CIs were also highly asymmetric, such that the selective power was on average not negatively impacted. In contrast, PoSI intervals (Lasso-CV-PoSI, ALasso-CV-PoSI) are symmetric by definition and were narrower than the SI CIs but they tended to be the widest of all inference procedures for strong, true predictors. The ALasso-CV-SI and ALasso-Neg-SI intervals were generally narrower and less prone to extreme widths than the corresponding CIs for Lasso-CV-SI and Lasso-Neg-SI, likely due to the ability of the adaptive Lasso to select relevant predictors more accurately. If runs with unstable CIs were removed from the analysis, the width and variability of the selective CIs for the SI method decreased noticeably (not shown).

#### Stability

The Lasso-CV-SI method sometimes produced intervals with infinite confidence limits, or which did not include the point estimate of the regression coefficient. The CI widths were generally extremely sensitive to the inclusion of weak or false predictors. When such predictors were selected by chance, small changes in the coefficient estimates led to large changes in the CI widths, i.e. highly variable CIs, potentially even for other, stronger predictors. We therefore termed the CIs in such situations as “unstable”. Almost 26% of the iterations of the realistic setup resulted in unstable CIs, while this happened for only 5% of the runs in the smaller toy setup. This issue was most prevalent when all predictors of the full model were strongly positively correlated and its prevalence generally increased with the signal-to-noise ratio or sample size due to the Lasso’s tendency to include weak predictors. The problem was much less severe for methods with less frequent false selections, such as the ALasso-CV-SI, Lasso-Neg-SI and ALasso-Neg-SI (each with less than 7% of all simulation iterations with unstable CIs, respectively).

### Model and variable selection

Generally, the adaptive Lasso was better able to identify the true model and led to fewer false selections in both simulation setups, in particular with higher signal-to-noise ratios. In contrast, the false positive rate increased for Lasso-CV with higher target R^2^. Lasso-Neg and ALasso-Neg generally led to very sparse models, with good properties regarding false positives, but at a higher probability of missing important predictors. Lasso-CV-Split and ALasso-CV-Split resulted in slightly lower accuracy of model selection due to less data being available for the selection. Results from both simulation setups are summarised in Supplementary Figures S9 and S10, with a detailed view per variable given in Supplementary Figure S11.

### Predictive accuracy

**Fig. 4:**
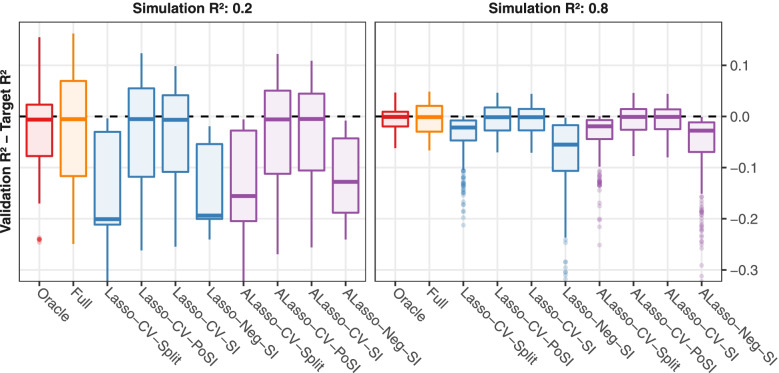
Toy simulation study: predictive accuracy in terms of difference of validation R^2^ and target simulation R^2^. The target simulation R^2^ was 0.2 in left panel, 0.8 in right panel. For each scenario, predictive accuracy was estimated by simulation, and over all scenarios, the values were summarised by boxplots. Dashed lines mark an optimal difference of zero. Colors indicate the type of variable selection. Results for the realistic setup are comparable (Supplementary Figure S12)

Predictive accuracy on validation data is depicted in Fig. 4 and Supplementary Figure S12. With increasing sample size, the target R^2^ values were achieved by the reference methods Full and Oracle, and most methods tuned by CV (Lasso-CV-SI, Lasso-CV-PoSI, ALasso-CV-SI, ALasso-CV-PoSI). However, Lasso-Neg-SI and ALasso-Neg-SI often led to very sparse models, resulting in inferior predictive accuracy. Similarly, for Lasso-CV-Split and ALasso-CV-Split only half the data was available to estimate effects and make predictions. This sub-optimal trade-off is clearly noticeable in the results.

### Summary of main simulation results

A high-level overview of the results is provided in Table 4. The Lasso-CV-Split and ALasso-CV-Split approaches led to generally acceptable coverage properties of the CIs even for weak predictors (i.e. within simulation variability for most scenarios), but this came at the cost of diminished predictive performance and statistical efficiency. The Lasso-CV-SI and ALasso-CV-SI approaches delivered acceptable results within expected simulation variability, but especially the latter could not guarantee nominal coverages. In particular, coverage was too low for variables with selection frequencies lower than 50%. Also, these methods suffered from highly variable CI widths, in particular when the Lasso “just barely” included certain variables with a very small penalized coefficient, leading to issues in the computation of the CIs [33]. Lasso-Neg-SI and ALasso-Neg-SI led to slightly improved properties of the CIs, as well as lower probability to include weak predictors, but had a clear negative impact on prediction performance. The Lasso-CV-PoSI and ALasso-CV-PoSI methods led to extremely conservative CIs with coverage consistently higher than the chosen level of significance and with very low selective type 1 error. Therefore, these methods may be too conservative to be used as default inference method for the Lasso. They may be applied if very strict error control is desired, potentially even stricter than indicated by the chosen level of significance, and only a moderate number of candidate predictors are of interest (e.g. a pool of 25 variables to be selected from) due to their computational demand (Supplementary Material Sect. 4.1). Alternatively, the PoSI methods may be useful when only a subset of variables, e.g. a single predictor of interest, is to undergo selection.

**Table 4:** Overview of main results for the primary estimands of our simulation study

Method	Stability	Coverage	Power	Type 1 error
Lasso_CV_Split	No concern	Acceptable	Low	Acceptable
Lasso_CV_PoSI	No concern	Too high	Low	Low
Lasso_CV_SI	Problematic	Acceptable	Acceptable	High
Lasso_Neg_SI	Problematic	Acceptable	Acceptable	Acceptable
ALasso_CV_Split	No concern	Acceptable	Acceptable	Acceptable
ALasso_CV_PoSI	No concern	Too high	Acceptable	Acceptable
ALasso_CV_SI	Problematic	Too low	High	High
ALasso_Neg_SI	Problematic	Acceptable	High	High

By “Acceptable” we mean that results were mostly (i.e. in median over all scenarios) within the expected simulation variability

### Extensions of the main simulation design

Beyond the results reported in Table 4, our study admits some further insights from the extended simulation setups. First, the results on 90% selective CIs were corroborated by simulations with 95% selective CIs (results not shown). In the second extension setup we found that while the selected penalization parameters differ, the properties of selective CIs using Lasso with CV, AIC and BIC were comparable (see Supplementary Figure S13). Lastly, in the third extension setup we found that the conclusions for logistic regression were similar to those presented in Table 4 (see Supplementary Figure S14). However, the PoSI method was not yet available for logistic regression and was excluded here.

## Real data example

We use Johnson’s body fat dataset [52] of 252 men to demonstrate a practical application of the selective inference framework. Following Johnson’s original publication, one observation was removed from the analysis due to implausible values. The dataset is freely available at the original article's website [53].

### Research question

The research question was to develop a prediction model for the percentage of bodyfat, measured by underwater weighting according to Siri’s formula, using multiple linear regression [54]. The candidate predictors were age (in decades), height (dm), weight (kg) and ten anthropometric measurements (all in cm). The goal of variable selection in this case study was to optimize the number of measurements necessary for the body fat estimation in future applications, rather than studying their causal relationship with the outcome.

### Analysis

We analyzed the dataset with the methods of our study (Table 2). In-line with recommendations on practical applications of variable selection, we additionally computed variable selection frequencies using 100 subsampling resamples of the dataset [2, 55].

### Results

An interesting feature of the dataset is its blocked correlation structure: the body measurements and weight are highly correlated (mean pairwise Pearson correlation of 0.65 between the individual variables), while age and height are rather uncorrelated (mean pairwise Pearson correlation with all other variables of 0.03 and 0.28, respectively). Therefore, even variables excluded from the final model cannot necessarily be deemed as “not predictive”, since they are often correlated to a “predictor” in the final (sub)model. It is therefore natural to be interested in inference about the specific set of selected variables, rather than targeting the full model. In the latter case, any assumption about the correctness of the chosen submodel would be questionable due to the high correlations.

**Fig. 5:**
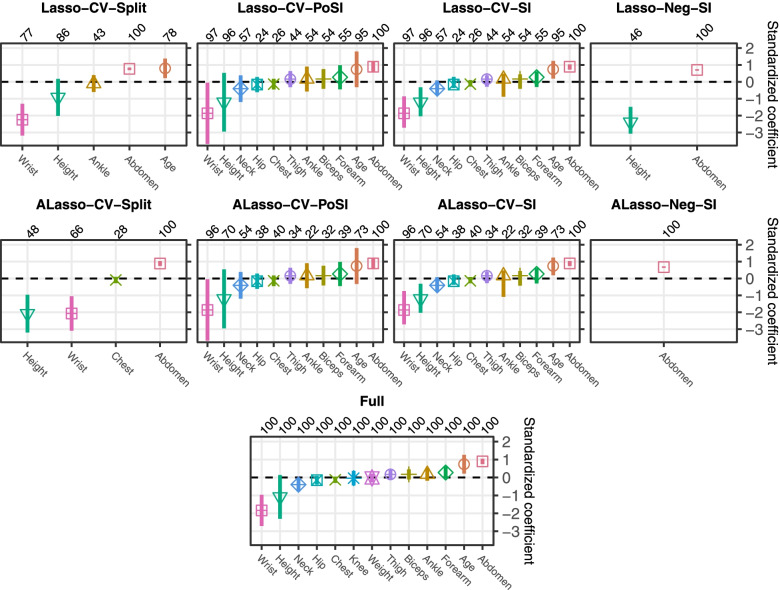
Real data example: point estimates and 90% selective CIs for regression coefficients. Results are shown at the original scales of the variables. Each method is depicted in a separate panel. The variables are ordered by increasing standardized coefficients. The individual selection frequencies estimated by 100 subsamples are given as percentages above each panel

Variable selection frequencies of the selected variables were mostly above 50%, and for variables with lower frequencies, the CIs had reasonable widths. Figure 5 provides a comparative presentation of the results, although in a real analysis each method would be interpreted by itself. As an example, assume the statistical analysis plan outlined the use of the Lasso tuned by CV, followed by SI for selective inference (Lasso-CV-SI). This procedure selected eleven variables, out of which four (abdomen, wrist, age and height) had corresponding 90% CIs excluding zero, and seven CIs including zero. All CIs included the respective point estimates and had finite limits. All variables with CIs excluding zero had resampling selection frequencies of 95% or more, while the variables with CIs including zero had frequencies of 57% or less. Some CIs were very asymmetric. In comparison, the Lasso-CV-PoSI approach led to wider CIs that excluded zero only for abdomen and wrist. These different results probably reflect different use-cases: the SI method could be potentially useful in early phase, explorative stages to single out weak predictors surviving the Lasso screening; the PoSI method could be favoured in later phase research where false positives are of particular concern and inference should respect all kinds of selection mechanisms during analysis. If the ALasso was chosen for variable selection, the resulting models were similar to the Lasso if CV was used, but sparser with sample splitting or the Neghaban methods to select the penalty parameter.

## Discussion

In this work, we studied the properties of proposals for selective inference for the Lasso available as R software packages via a neutral comparison study. In actual applications, selective inference requires careful consideration of the intended use of a statistical model by the practitioner. For example, in descriptive or predictive research, the interest often lies in the finally selected model, rather than in addressing the population parameters of an assumed underlying data generating mechanism. In explanatory research, the data-adaptive selection of confounders may provide benefits in terms of reduced mean squared error when estimating the exposure effect, given the selected submodel is sufficient to control for all confounders or confounding is addressed by specialized methods such as inverse probability weighting. Selective inference is well suited for these usages, where it is not of primary concern whether a “true model” exists and what its parameters are. Through accounting for the selection of the model of interest, over-optimism via false positive selections in reported inference can be reduced, thereby enhancing replicability.

This was elaborated in our real data example, where an additional inference step after selection allowed to single out variables which were likely most relevant to the prediction of the outcome, in contrast to those with small, but non-zero point estimates. This is of great interest when using the Lasso, as it is known to have a high probability for the inclusion of weak predictors. However, selective inference does not come free, as there is not only a trade-off regarding power and type 1 error, but also a relation of selection and inference accuracy. As demonstrated in our simulations, the SI approach could only provide reliable results when the selected submodel was stable in the first place, i.e. only included variables with comparatively high selection frequency.

In this study we made extensive use of the software packages in R that are available to conduct selective inference for the Lasso, *PoSI* and *selectiveInference* [48, 49]. While we found the functions in these packages purposeful and sufficiently well developed for use in our simulation study, there is still a lack of smooth integration into the common framework of modelling functions in R such as *lm*, *glm, step* or *glmnet*. Given that the methodology of selective inference is rather new, providing easily accessible software and a satisfactory user-experience may increase the adoption of the methods by applied statisticians and their collaborators.

Our study has some limitations. It was restricted to recent approaches to selective inference with a focus on the submodel view of inference. The data generating mechanism of our simulations was chosen to be sparse and all effects were assumed linear. We used mostly default parameters for the implementation of the methods if they were sensible, ensuring comparability between the methods. Lastly, for the PoSI and SI methods we derived an estimate for the outcome variance from the residual variance of the full model, which is in line with the literature and often the only estimate available in practical applications.

Based on our extension studies, we expect the findings from our simulations to transfer to other confidence levels (e.g. 95%), Lasso tuning based on AIC or BIC, and logistic regression.

## Conclusions

In general, we recommend combining post-selection inference with an assessment of model stability and variable selection frequencies. We found that the SI methodology for selective inference yielded acceptable actual coverage rates in most scenarios. It is therefore our recommended approach for most cases. If the number of observations is large and simplicity is strongly favoured over efficiency, then the Split approach, or a more refined variant (data carving [11, 30]), are an alternative. If only few predictors undergo variable selection (i.e. up to 5) or the avoidance of false positive claims of significance is a concern, then the conservative PoSI approach may be useful. For the adaptive Lasso, especially if the penalization strength is tuned, the SI approach should be avoided because of violation of the claimed confidence levels. Selective inference is a useful tool for regression modelling using the Lasso, but its application in practice requires sophisticated interpretation and awareness of the intended use of the model, as well as the availability of more user-friendly software.

## Supplementary Information

**Additional file 1. **

## Data Availability

The real-world dataset analyzed during the current study is available from http://jse.amstat.org/v4n1/datasets.johnson.html. Code to process the dataset as in the real data example, and to generate the datasets analyzed in the simulation study is provided in a repository at https://github.com/matherealize/LassoSI.

## Abbreviations

AIC: Akaike information criterion
ALasso: Adaptive Lasso
BIC: Bayesian information criterion
CI: Confidence interval
CV: Cross-validation
Neg: Neghaban et al. method to tune penalization
PoSI: Post-selection inference, method by Berk et al.
SI: Selective inference conditional for the Lasso, method by Lee et al.
