# Supplementary material for: Evaluating methods for Lasso selective inference in biomedical research: a comparative simulation study
Source: BMC Med Res Methodol. 2022 Jul 26;22:206. doi: 10.1186/s12874-022-01681-y (PMC9316707; doi:10.1186/s12874-022-01681-y)
Supplement: Supplementary file 1 — Additional file 1. [file 12874_2022_1681_MOESM1_ESM.pdf]

Supplementary material
Evaluating methods for Lasso selective inference in biomedical research: a comparative simulation study

Michael Kammer[1,2], Daniela Dunkler[1], Stefan Michiels[3] and Georg Heinze[1,*]

1 Medical University of Vienna, Center for Medical Statistics, Informatics and Intelligent Systems, Section for Clinical Biometrics, Vienna, Austria

2 Medical University of Vienna, Department for Internal Medicine III, Division of Nephrology and Dialysis, Vienna, Austria

3 Service de Biostatistique et d'Epidémiologie, Gustave Roussy; INSERM, CESP U1018, University Paris-Saclay, Villejuif, France

* Corresponding author:

Medical University of Vienna
Center for Medical Statistics, Informatics and Intelligent Systems
Section for Clinical Biometrics
Spitalgasse 23
1090 Vienna
Austria

georg.heinze@meduniwien.ac.at

1. Views on post-selection inference

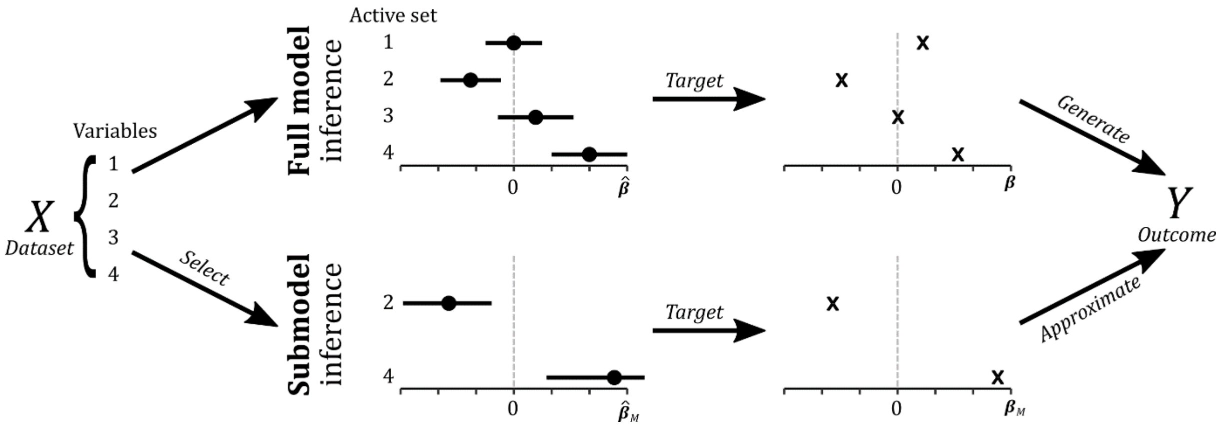

Supplementary Figure S 1: Graphical illustration of two views of post-selection inference. Berk and colleagues (1) introduced two alternative views on post-selection inference. In the full model view, the population parameters of interest are those of the full set of candidate predictors, possibly including interactions or non-linear terms. The "full" model comprising all candidate predictors is the object of interest for future research and is assumed to be a description of the data generating mechanism. Variable selection therefore merely amounts to forcing some of the coefficients in the estimated model to zero, but a corresponding population parameter still exists and serves as the target of post-selection inference. In the alternative submodel view, interest lies in the parameters of the selected variables only. The full model does not have a special meaning, as a models' primary purpose is to provide a succinct description of the association of the outcome and the independent variables, not necessarily capturing the data generating mechanism. The hypotheses to be assessed, and the population quantities, therefore depend on the selected submodel.

A submodel M of the full model can be interpreted as a linear approximation to the full model with no requirements regarding the correctness of its parameters, i.e. that its coefficients are the same as for the full model. In fact the full model and submodel targets differ unless there is no correlation, and interval estimates, indicated by black lines, are different (likely wider) between full model and submodel inference due to the need to account for variable selection.

2. Simulation profile

This brief overview provides a high-level summary and pointers to the essential definitions of the simulation study in this manuscript.

2.1. Design

Aim: To evaluate recent proposals for selective inference in the context of Lasso regression regarding their frequentist properties in practical usage scenarios.

Data generation: Aimed at studying different correlation patterns and effect distribution in sparse settings. The data generation procedure is outlined in the figure below.

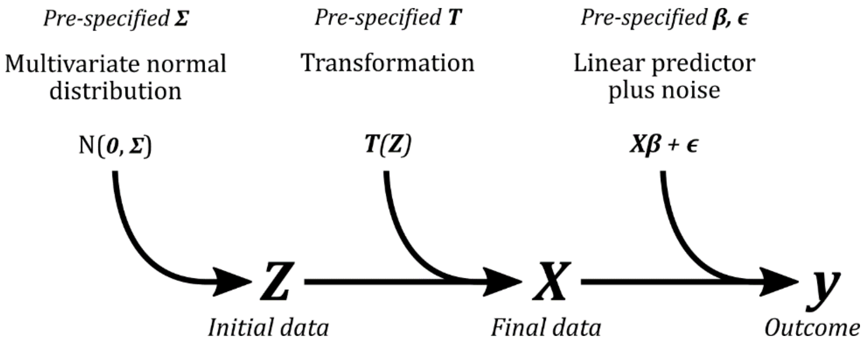

Two setups with different pre-specifications (notation referencing figure above):

		**Toy setup**	**Realistic setup**
**Fixed design parameters**	Motivation	Simplicity, insight	Realistic data
	Regression type	Gaussian	Gaussian
	Number of variables	4	17
	Type of variables	Continuous	Continuous, binary
	Distribution of variables	Gaussian	Mixed (specified in Supplementary Table S 2)
**Varying design parameters**	Correlation structures $\boldsymbol{\Sigma}$	7 blocked correlation matrices with no or strong correlation	Fixed, mimicking real study (Supplementary Figure S 3)
	Coefficient structures $\boldsymbol{\beta}$	10 (Supplementary Table S 1)	13 (Supplementary Table S 3)

	True target R^2 (noise ϵ)	0.2, 0.5, 0.8	0.2, 0.5, 0.8
	Observations per variable	5, 10, 50	5, 10, 50
**Simulation parameters**	Number of scenarios	630	117
	Iterations per scenario	900	900

Primary estimands: selective coverage, power and type 1 error (see Table 3)
Further estimands: true model and variable selection frequencies, width of confidence intervals, prediction accuracy, inference stability.

Methods: Lasso and adaptive Lasso for variable selection, tuned with either cross-validation (CV) or using a fixed estimated penalization strength following Negahban et al (2). Sample splitting, or the approaches by Berk et al (1) and Lee et al (3) for selective inference (see Table 2).

Performance measures: Difference to nominal significance level for selective coverage and type 1 error. Highest value for selective power. Minimal median and interquartile range for width of confidence intervals. Difference of validation R^2 and true R^2 for prediction accuracy.

2.2. Coding and execution

Study conducted in R version 3.5.1. using the packages glmnet (version 2.0-18) (4), selectiveInference (version 1.2.4) (5) and PoSI (version 1.0) (6). Data was generated using the simdata package (version 0.5.0.9000) (7).

2.3. Results

Primary estimands: Selective coverage and type 1 error mostly acceptable for all inference approaches for Lasso, undercoverage for adaptive Lasso tuned by CV for the Lee et al method; selective power generally lower for Berk et al approach but very low type 1 error (Figure 1, Figure 2, Supplementary Figure S 5).

Width of confidence intervals: Asymmetric and highly variable for Lee et al method, generally lower variability with adaptive Lasso. Symmetric and robust for Berk et al method (Figure 3, Supplementary Figure S 8).

Stability of inference: Method by Lee et al after CV tuned Lasso provided confidence intervals not including the point estimate or having infinite width in a non-negligible fraction of iterations

(30% for real setup, 5% for toy setup). Issue less severe for adaptive Lasso (7% and 1% respectively).

True model and variable selection: Adaptive Lasso more often selects true data generating model and less often includes false positives than Lasso (Supplementary Figure S 10, Supplementary Figure S 11).

Prediction performance: Methods using split-sample and Neghaban et al had lower prediction performance compared to other methods (Figure 4, Supplementary Figure S 12).

3. Simulation study details

Code to generate the datasets used in this study is available from https://github.com/matherealize/LassoSI.

3.1. Derivation of estimators

We present the derivation of our simulation approximation of the selective estimands exemplary for the case of selective coverage after a certain variable selection procedure of interest. Let the model $M \subseteq M_F = \{1, \dots, p\}$ be fixed and let $j \in M$. We denote the complete set of simulation iterations as $S = \{1, \dots, n_{sim}\}$ for a fixed total number of simulations n_{sim}. As in the main manuscript, we use the notation $\widehat{M}$ to indicate the random variable representing the model selected by the variable selection procedure. By the use of $\widehat{M}_s$ we denote the model chosen in a specific iteration s of the simulation study.

In each iteration s of the simulation study in which the selected model $\widehat{M}_s$ coincides with M, we observe the event if a selective CI for i covers its target parameter or not (if $\widehat{M}_s \neq M$ inference is neither available, nor of interest to us). From this we can estimate the conditional coverage probabilities $\mathbb{P}\left[\beta_{j,M} \in CI_{j,M} \middle| \widehat{M} = M, j \in \widehat{M}\right] = \frac{\sum_{s \in S} \mathbb{I}\left[\widehat{M}_s = M \wedge \beta_{j,M} \in CI_{j,M}\right]}{\sum_{s \in S} \mathbb{I}\left[\widehat{M}_s = M\right]}$, where we use $\mathbb{I}$ to denote the indicator function. The formula can be interpreted as the number of all iterations where the CI covered its target parameter (provided it exists), divided by the frequency how often the specific model M was selected. If M was never selected, then the conditional coverage probability is estimated as zero, but it plays no role in the further computations. By the law of total probability we can use these probabilities to compute the conditional coverage probabilities for a fixed variable $j \in M_F$:

$$\mathbb{P}\left[\beta_{j,\widehat{M}} \in CI_{j,\widehat{M}} \middle| j \in \widehat{M}\right] = \sum_{M \subseteq M_F} \left(\mathbb{P}\left[\beta_{j,M} \in CI_{j,M} \middle| \widehat{M} = M, j \in \widehat{M}\right] \mathbb{P}\left[\widehat{M} = M \middle| j \in \widehat{M}\right]\right).$$

The latter term $\mathbb{P}\left[\widehat{M} = M \middle| j \in \widehat{M}\right]$ for fixed M and j is estimated by $\frac{\sum_{s \in S} \mathbb{I}\left[\widehat{M}_s = M\right]}{\sum_{s \in S} \mathbb{I}\left[j \in \widehat{M}_s\right]}$. Note that for models M which do not contain the variable of interest, $\mathbb{P}\left[\widehat{M} = M \middle| j \in \widehat{M}\right] = 0$ (i.e. in such cases

inference is not available).
For the overall expected selective coverage, we further marginalize over all candidate predictors.

$$\mathbb{P}\left[\beta_{.,\hat{M}} \in CI_{.,\hat{M}}\right] = \sum_{j \in M_F} \left(\mathbb{P}\left[\beta_{j,\hat{M}} \in CI_{j,M} \middle| j \in \hat{M}\right] \mathbb{P}\left[j \in \hat{M}\right]\right).$$

Note that the term $\mathbb{P}\left[j \in \hat{M}\right]$ can be derived from the variable selection frequencies $\frac{\sum_{s \in S} \mathbb{I}[j \in \hat{M}_s]}{n_{sim}}$ through re-normalisation by $1/n_{sim} \sum_{k \in M_F} \sum_{s \in S} \mathbb{I}\left[k \in \hat{M}_s\right]$ such that the result is a probability distribution over the candidate predictors, i.e. $\sum_{j \in M_F} \mathbb{P}\left[j \in \hat{M}\right] = 1$. The expression $\sum_{k \in M_F} \sum_{s \in S} \mathbb{I}\left[k \in \hat{M}_s\right]$ is simply counting the total number of selection events for the variable selection procedure over all iterations of the simulation scenario.
In practice, the computations simplify drastically, resulting in the estimators shown in Table 3. For example, the approximation of overall selective coverage can be explicitly obtained from the equations above as

$$\mathbb{P}\left[\beta_{.,\hat{M}} \in CI_{.,\hat{M}}\right] = \sum_{j \in M_F} \left(\sum_{M \subseteq M_F} \left(\mathbb{P}\left[\beta_{j,M} \in CI_{j,M} \middle| \hat{M} = M, j \in \hat{M}\right] \mathbb{P}\left[\hat{M} = M \middle| j \in \hat{M}\right]\right) \mathbb{P}\left[j \in \hat{M}\right] \right)$$

$$= \sum_{j \in M_F} \left(\sum_{M \subseteq M_F} \left(\frac{\sum_{s \in S} \mathbb{I}\left[\hat{M}_s = M \wedge \beta_{j,M} \in CI_{j,M}\right]}{\sum_{s \in S} \mathbb{I}\left[\hat{M}_s = M\right]} \cdot \frac{\sum_{s \in S} \mathbb{I}\left[\hat{M}_s = M\right]}{\sum_{s \in S} \mathbb{I}\left[j \in \hat{M}_s\right]} \right) \cdot \frac{\sum_{s \in S} \mathbb{I}\left[j \in \hat{M}_s\right]}{n_{sim}} \cdot \frac{n_{sim}}{\sum_{k \in M_F} \sum_{s \in S} \mathbb{I}\left[k \in \hat{M}_s\right]} \right)$$

$$= \sum_{j \in M_F} \left(\sum_{M \subseteq M_F} \left(\frac{\sum_{s \in S} \mathbb{I}\left[\hat{M}_s = M \wedge \beta_{j,M} \in CI_{j,M}\right]}{\sum_{s \in S} \mathbb{I}\left[j \in \hat{M}_s\right]} \right) \cdot \frac{\sum_{s \in S} \mathbb{I}\left[j \in \hat{M}_s\right]}{\sum_{k \in M_F} \sum_{s \in S} \mathbb{I}\left[k \in \hat{M}_s\right]} \right)$$

$$= \sum_{j \in M_F} \sum_{M \subseteq M_F} \left(\frac{\sum_{s \in S} \mathbb{I}\left[\hat{M}_s = M \wedge \beta_{j,M} \in CI_{j,M}\right]}{\sum_{s \in S} \mathbb{I}\left[j \in \hat{M}_s\right]} \cdot \frac{\sum_{s \in S} \mathbb{I}\left[j \in \hat{M}_s\right]}{\sum_{k \in M_F} \sum_{s \in S} \mathbb{I}\left[k \in \hat{M}_s\right]} \right)$$

$$= \sum_{j \in M_F} \sum_{M \subseteq M_F} \left(\frac{\sum_{s \in S} \mathbb{I}\left[\hat{M}_s = M \wedge \beta_{j,M} \in CI_{j,M}\right]}{\sum_{k \in M_F} \sum_{s \in S} \mathbb{I}\left[k \in \hat{M}_s\right]} \right),$$

which outlines significant simplifications and yields the result as given in Table 3 in the main manuscript. Note that the equation on the right hand can then be easily interpreted as "evaluate whether a CI covers its target parameter, whenever a CI is available".

In the case of a conditional quantity, i.e. conditional on a specific variable of interest being selected, the analogous interpretation would be "evaluate whether a CI for variable j covers its target parameter, whenever a CI for j is available (i.e. j was selected)".

3.2. Toy simulation setup

The toy setup was kept extremely simple with four standard normal distributed candidate predictors in order to provide a focused assessment of the methods in our study. The rationale was that more variables just meant more (hard to control) noise and a larger number of essentially equivalent ways to distribute effects, blurring the conclusions and making the results less intelligible. Using this distilled setup allowed us to study a variety of 7 different structures for the true correlation matrix:

- Uncorrelated (1 matrix design): all variables were uncorrelated
- Correlated (2 matrix designs): all variables were equally correlated (either 0.8 or -0.3).
- Two 2x2 blocks (2 matrix designs): the correlation matrix consisted of two blocks of size 2x2. Correlation within the blocks either was the same (0.8) or mixed (0.8 / -0.8 respectively). There was no correlation between the blocks of variables.
- One 3x3 block (2 matrix designs): the correlation matrix consisted of two blocks, one of size 1x1 (i.e. a single variable) and one of size 3x3. The last 3 variables were equally correlated (either 0.8 or -0.45). There was no correlation between the blocks of variables.

All used correlation values were chosen rather high in order to have a strong impact on the results. We defined 10 possible structures for the vector of true regression coefficients $\boldsymbol{\beta} = (\beta_1, \beta_2, \beta_3, \beta_4)$, with (standardized) effect strengths of either 1 or 0.1. These are listed in Supplementary Table S 1. We used a full factorial design, resulting in 630 scenarios for this toy setup. Each individual simulation scenario comprised 900 repetitions. Example code demonstrating how the data was simulated using the simdata package (7) is provided in the file *Toy_Setup_Demo.R* in the online code repository.

Supplementary Table S 1: Overview of coefficient structures in toy simulation setup.

Number	Coefficients	Remarks
1	$\beta_1 = 1$	
2	$\beta_1 = \beta_2 = 1$	
3	$\beta_1 = 1, \beta_2 = 0.1$	strongly differential effect size, Lasso is known to have difficulty dealing with such situations
4	$\beta_1 = \beta_2 = \beta_3 = \beta_4 = 1$	non-sparse situation
5	$\beta_1 = \beta_3 = 1$	differences to 2. due to block correlation structure
6	$\beta_1 = 1, \beta_3 = 0.1$	differences to 3. due to block correlation structure
7	$\beta_1 = 0.1, \beta_3 = 1$	differences to 3. and 6. due to block correlation structure
8	$\beta_3 = 1$	differences to 1. due to block correlation structure

9	$\beta_3 = \beta_4 = 1$	differences to 2. and 5. due to block correlation structure
10	$\beta_3 = 1, \beta_4 = 0.1$	differences to 6. due to block correlation structure

Only non-zero coefficients are specified on a standardized scale.

3.3. Realistic simulation setup

The realistic setup featured a fixed, complex correlation structure and different kinds of variable distributions based on real clinical data as presented in (8). After drawing standard normal data using a pre-specified correlation matrix (see Supplementary Figure S 2), the final simulated dataset was obtained by applying transformations according to Supplementary Table S 2. The 17 final predictors comprised continuous and discrete variables, with clusters of highly correlated variables as well as uncorrelated ones (multiple correlation coefficients between 0 and 0.7). We considered 13 different options for the vector of standardized regression coefficients = $(\beta_i)_{i=1,\dots,17}$, based on the correlation network to provide the simulation with interesting correlation scenarios. The values are listed in Supplementary Table S 3. In this setup, a full factorial design led to 117 scenarios.

This setup was based on the design presented in (8). Example code demonstrating how the data was simulated using the simdata package (7) is provided in the file *Realistic_Setup_Demo.R* in the online code repository.

Correlation structure

The correlation matrix used for drawing data from a multivariate normal distribution was modified from the original to feature stronger correlations. The following network depicts the final correlation structure. Individual variables as nodes in a graph, correlations between two variables are indicated as an edge with specified correlation coefficient.

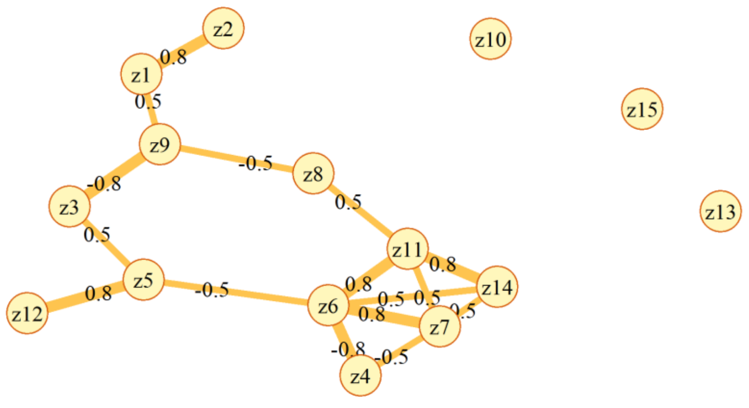

Supplementary Figure S 2: Initial correlation network for real simulation setup.

Data transformation

Data from the initial multivariate distribution was transformed to achieve different variable distributions. The transformations were the same as in the original publication:

Supplementary Table S 2: Final variables for real simulation setup.

**Initial variable**	**Final variable**	**Type**	**Multiple correlation**
z_1	$v_1 = [10z_1 + 55]$	Continuous	0.63
z_2	$v_2 = I(z_2 < 0.6)$	Binary	0.58
z_3	$v_3 = \exp(0.4z_3 + 3)$	Continuous, skewed	0.52
z_4	$v_4 = I(z_4 \geq -1.2)$	Ordinal	0.44
z_4	$v_5 = I(z_4 \geq 0.75)$	Ordinal	0.35
z_5	$v_6 = \exp(0.5z_5 + 1.5)$	Continuous, skewed	0.68
z_6	$v_7 = [\max(0, 100\exp(z_6) - 20)]$	Continuous, skewed	0.66
z_7	$v_8 = [\max(0, 80\exp(z_7) - 20)]$	Continuous, skewed	0.65
z_8	$v_9 = I(z_8 < -0.35)$	Binary	0.43
z_9	$v_{10} = I(0.5 \leq z_9 < 1.5)$	Ordinal	0.46
z_9	$v_{11} = I(1.5 \leq z_9)$	Ordinal	0.47
z_{10}	$v_{12} = 0.01[100(z_{10} + 4)^2]$	Continuous	0.01
z_{11}	$v_{13} = [10z_{11} + 55]$	Continuous	0.71
z_{12}	$v_{14} = [10z_{12} + 55]$	Continuous	0.63
z_{13}	$v_{15} = [10z_{13} + 55]$	Continuous	0.01
z_{14}	$v_{16} = I(z_{14} < 0)$	Binary	0.61
z_{15}	$v_{17} = I(z_{15}10)$	Binary	0.01

Multiple correlation was estimated from a simulated dataset with 100000 observations.

The final correlation network was similar to the original one.

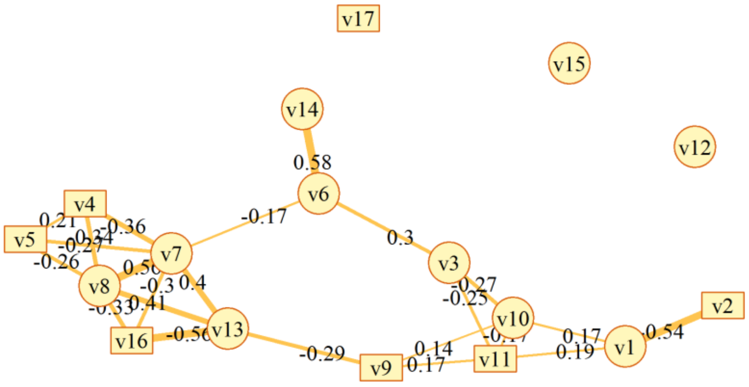

Supplementary Figure S 3: Final correlation network for real simulation setup. Discrete (binary, ordinal) variables are depicted as squares, continuous ones as circles. Correlations were estimated from a simulated dataset with 100000 observations.

Coefficients

Supplementary Table S 3: Overview of coefficient structures in real simulation setup.

Number	Coefficients	Remarks
1	$\beta_2 = \beta_4 = \beta_{14} = 1$	3 rather independent variables far apart in the network
2	$\beta_7 = \beta_8 = \beta_{13} = 1$	3 tightly clustered variables
3	$\beta_7 = \beta_8 = \beta_{13} = \beta_4 = \beta_5 = \beta_{16} = 1$	6 highly correlated variables
4	$\beta_7 = \beta_8 = \beta_{13} = 1,$ $\beta_4 = \beta_5 = \beta_{16} = 0.1$	weaker effect for one cluster of variables
5	$\beta_7 = \beta_8 = \beta_{13} = 0.1,$ $\beta_4 = \beta_5 = \beta_{16} = 1$	weaker effect for one cluster of variables
6	$\beta_7 = \beta_8 = \beta_{13} = 1,$ $\beta_4 = \beta_5 = \beta_{16} = -1$	negative effect for one cluster of variables
7	$\beta_7 = \beta_8 = \beta_{13} = -1,$ $\beta_4 = \beta_5 = \beta_{16} = 1$	negative effect for one cluster of variables
8	$\beta_7 = \beta_8 = \beta_{13} = \beta_2 = \beta_4 = \beta_{14} = 1$	weakly and strongly correlated variables mixed
9	$\beta_7 = \beta_8 = \beta_{13} = 1,$ $\beta_2 = \beta_4 = \beta_{14} = 0.1$	weakly and strongly correlated variables mixed with mixed effects
10	$\beta_7 = \beta_8 = \beta_{13} = 0.1,$ $\beta_2 = \beta_4 = \beta_{14} = 1$	weakly and strongly correlated variables mixed with mixed effects
11	$\beta_7 = \beta_8 = \beta_{13} = 1,$ $\beta_2 = \beta_4 = \beta_{14} = -1$	weakly and strongly correlated variables mixed with mixed effects

12	$\beta_7 = \beta_8 = \beta_{13} = -1,$ $\beta_2 = \beta_4 = \beta_{14} = 1$	weakly and strongly correlated variables mixed with mixed effects
13	$\beta_7 = \beta_8 = \beta_{13} = \beta_4 =$ $\beta_5 = \beta_{16} = \beta_2 = \beta_{14} = 1$	mixed scenario

Only non-zero coefficients are specified on a standardized scale.

3.4. Tuning of the penalization parameter $\boldsymbol{\lambda}$

- Cross-validation (CV): use 10-fold cross validation to obtain the penalization parameter with minimal estimated prediction error. The observed outcomes $\boldsymbol{y}$ directly affect the determined λ, which is therefore a random variable itself.
- Estimation following Negahban et al (Neg, (2)): this procedure relies on the observed data $\boldsymbol{X}$ and an estimate of the marginal outcome variance σ^2, and is therefore not specific to the observed outcomes $\boldsymbol{y}$. The penalization parameter is estimated through $\lambda = 2\mathbb{E}\left(||\boldsymbol{X}^T\epsilon||_\infty\right)$, where $\epsilon \sim N(0, \sigma^2)$. Thus, λ can be considered as a fixed, non-random parameter. This method was used only in combination with SI. We approximated the expectation required for the estimation of λ by 1000 internal simulations, using $\epsilon \sim N(0, \hat{\sigma}^2)$.

3.5. R package details

The algorithm implemented in the selectiveInference package involved a grid search for the bounds of the selective CIs for which the search space was extended from the default values to the interval [-1000, 1000] with 1000 interval steps. As a side note, some minor bugs were fixed in the package to ensure correct computation of the CIs. These concern logistic regression and have also been reported as issues on the package's Github repository.

- Confidence intervals for logistic regression may have the wrong sign. After studying the code, we believe this happens when the sign of the coefficient is negative, in which case the interval needs to be flipped.
- The passed significance level for logistic regression is ignored. A simple fix for this was done in the software used for this simulation study.

The algorithm for the computation of the PoSI constant as implemented in the PoSI package involved numeric simulations. Due to the computational burden, we reduced the number of these internal simulations to 500 for the realistic setup only.

4. Additional results

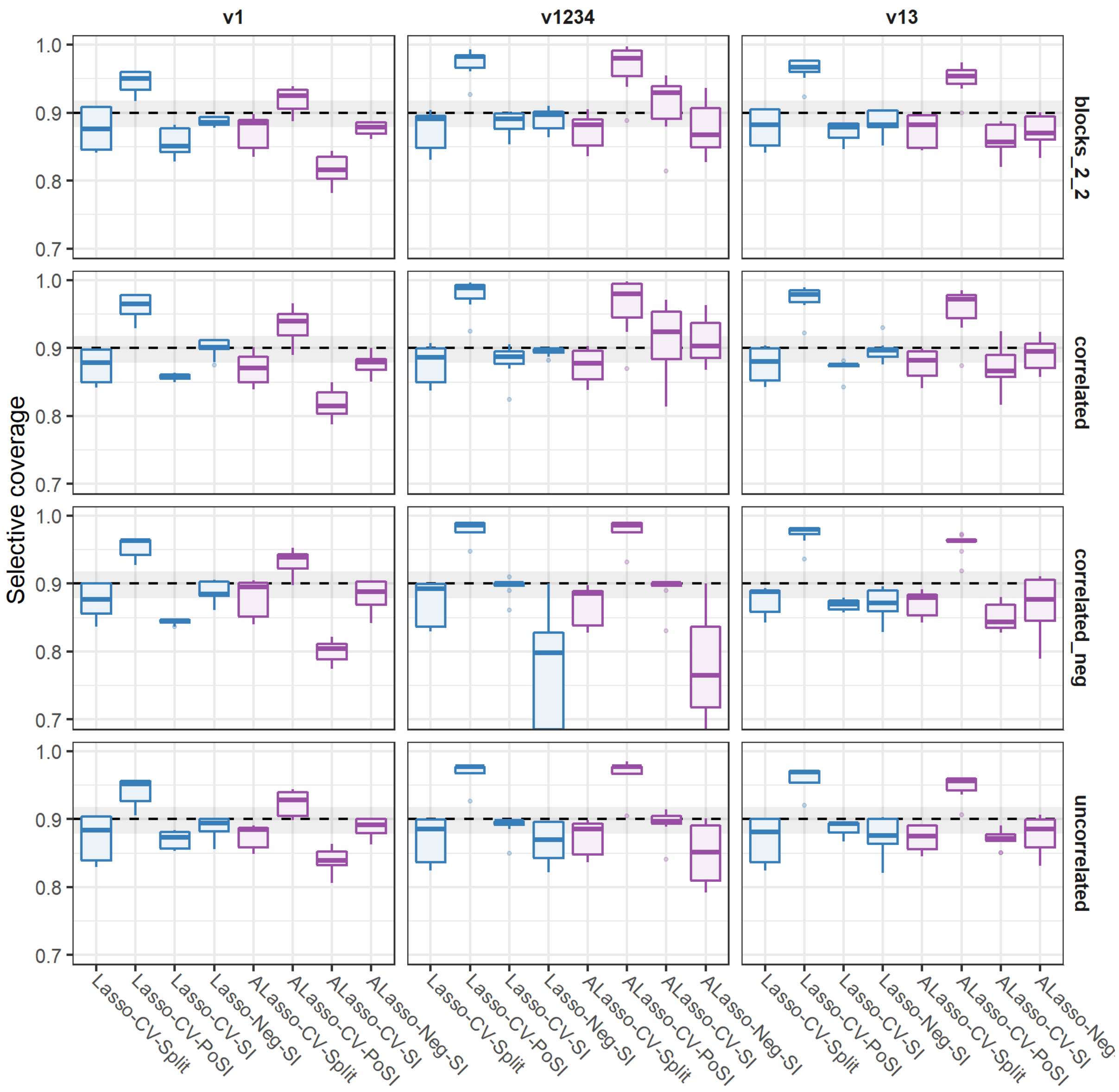

Supplementary Figure S 4: Toy simulation study: selective coverage from both toy simulation setup of selective 90% CIs for the submodel inference target stratified by correlation and coefficient structure. This figure provides a more in-depth view of the results in Figure 1 of the main manuscript. By comparing different panels, we demonstrate how the different methods behave in different scenarios. We show here a subset of all scenarios for two design factors of the simulation study (see Table 1 in the main manuscript), i.e. coefficient structure and correlation structure. The boxplots are summaries over the two remaining design factors, namely target R^2 and sample size and provide an insight into the variability of the results. The three coefficient structures shown are v1 – only the first variable has a true effect on the outcome; v1234 – all variables have a true effect on the outcome; v13 - the first and third variable have a true effect on the outcome; refer to Supplementary Table S 1 for exact

definitions. The correlation structures shown refer to block_2_2 – the true correlation matrix is a block matrix with two blocks of equal correlations; correlated – all variables have same true positive correlation; correlated_neg – all variables have same true negative correlation; uncorrelated – no true correlation between variables; refer to Supplement Section 3.2 for details. The nominal confidence level of 0.9 used in the construction of the CIs is depicted as dashed lines. Colors indicate the type of variable selection. Monte Carlo error is indicated by grey areas describing binomial 95% CIs expected at the nominal confidence level with 900 iterations.

In this plot, we can observe that the Split method yields similar results for all scenarios and for both Lasso and adaptive Lasso, demonstrating the robustness of the method. The PoSI method is shown to be conservative, which is demonstrated by e.g. the comparison of columns “v1” and “v1234”. Due to very wide CIs, the method has low selective power which is why the selective coverage is too high in column “v1234”, but closer to the nominal level in column “v1”. For the SI method it is also interesting to compare these columns. In contrast to PoSI, SI generally struggles with higher selective type 1 error, and thus the selective coverage is closer to nominal in column “v1234” than in column “v1”. This is particularly obvious when there are strong correlations between the variables, e.g .in rows “correlated” and “correlated_neg”. Lastly, the Neg method to tune the Lasso yields extremely sparse results, which translates to highly variable results for the different scenarios, but in particular in scenarios with negative corelations (row “correlated_neg”). Generally, the behaviour of all methods here is outlined in Table 4 in the main manuscript.

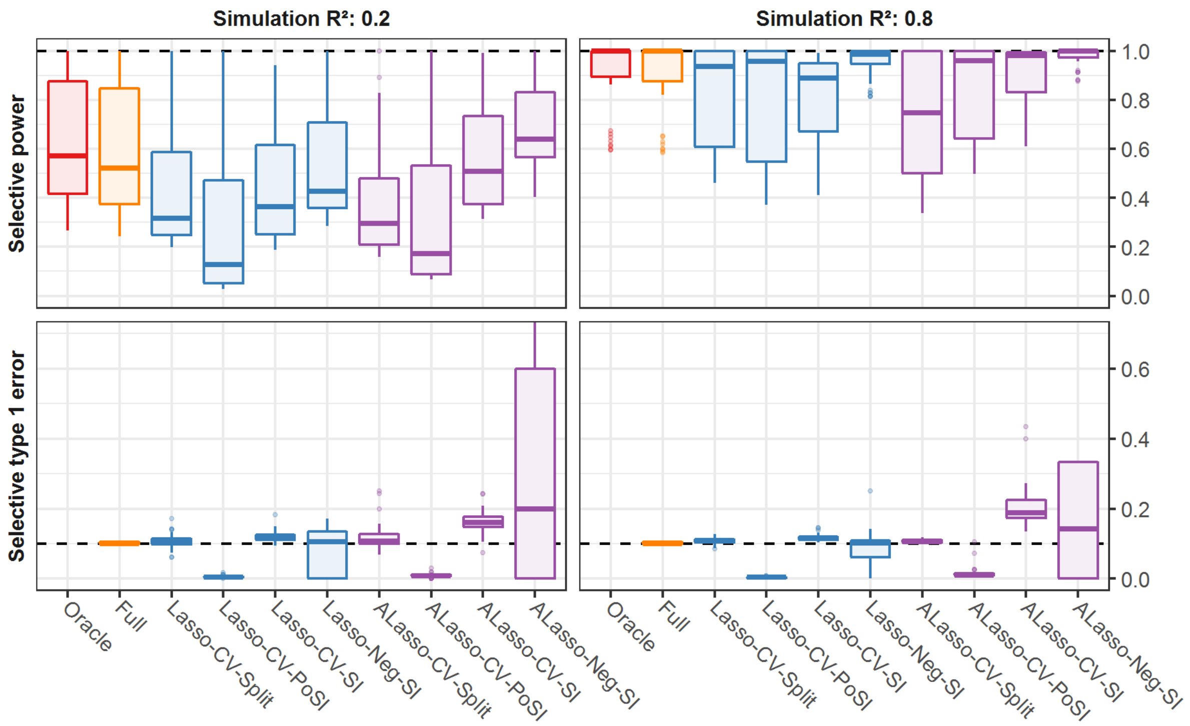

Supplementary Figure S 5: Realistic simulation study: selective power and selective type 1 error of selective 90% confidence intervals. For each scenario, power or type I error was estimated by simulation, and over all scenarios with specified target simulation R^2, the values were summarised by boxplots. The target values are depicted as dashed lines (1 for power, 0.1 for type 1 error). Colors indicate the type of variable selection.

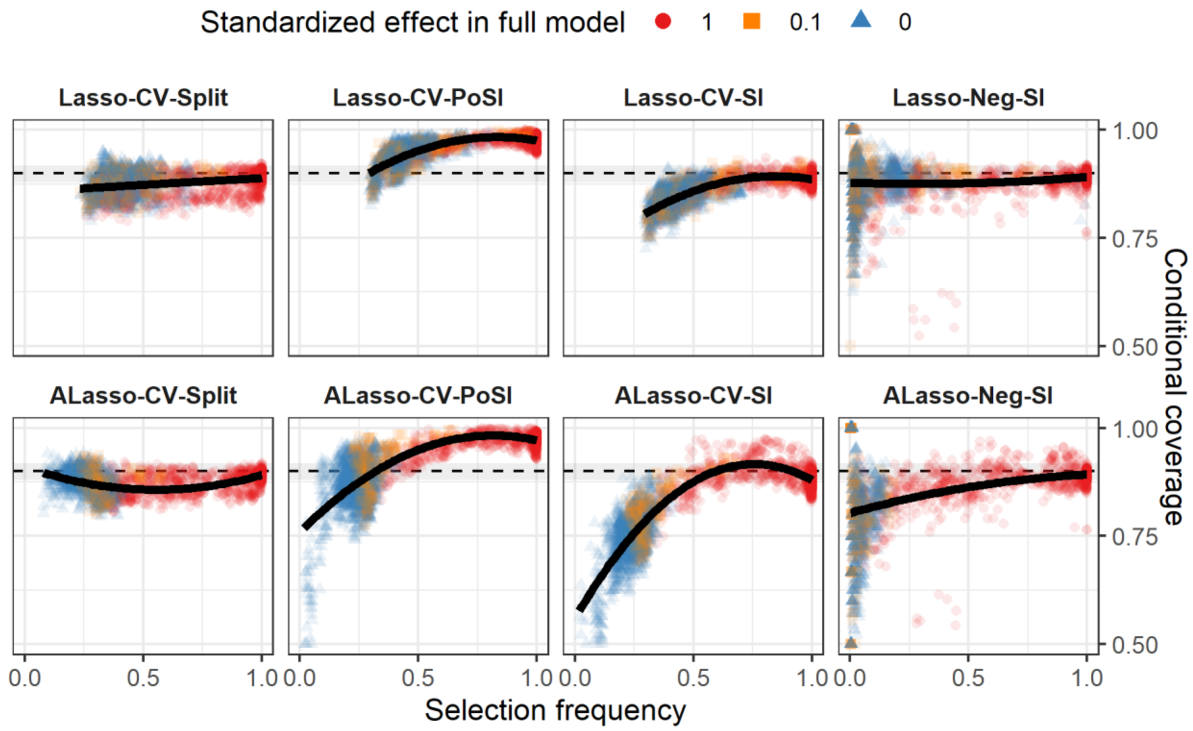

Supplementary Figure S 6: Toy simulation study: comparison of selection frequency and conditional selective coverage. Conditional selective coverage refers to the coverage probability for a specific, pre-specified variable of interest, in case it was selected into the model. An example would be when a new risk marker of interest is added to an existing set of predictors, but only considered for inference if it survives a variable selection procedure. In the plot, each dot represents results for a single variable in a specific simulation scenario. The target coverage value (0.9) is depicted as dashed line. Colors indicate if the variable is a predictor in the full model in the specific scenario. The black line provides a smoothed summary of the observed data (fitted with a quadratic B-spline term with 3 knots for selection frequency). Monte Carlo error is indicated by grey areas describing binomial 95% CIs expected at the nominal confidence level with 900 iterations. Ideally, the black line would be straight and on top of the dashed line at 0.9. The results indicate that the conditional coverage decreases with decreasing variable selection probability for the PoSI and SI methods with CV tuning.

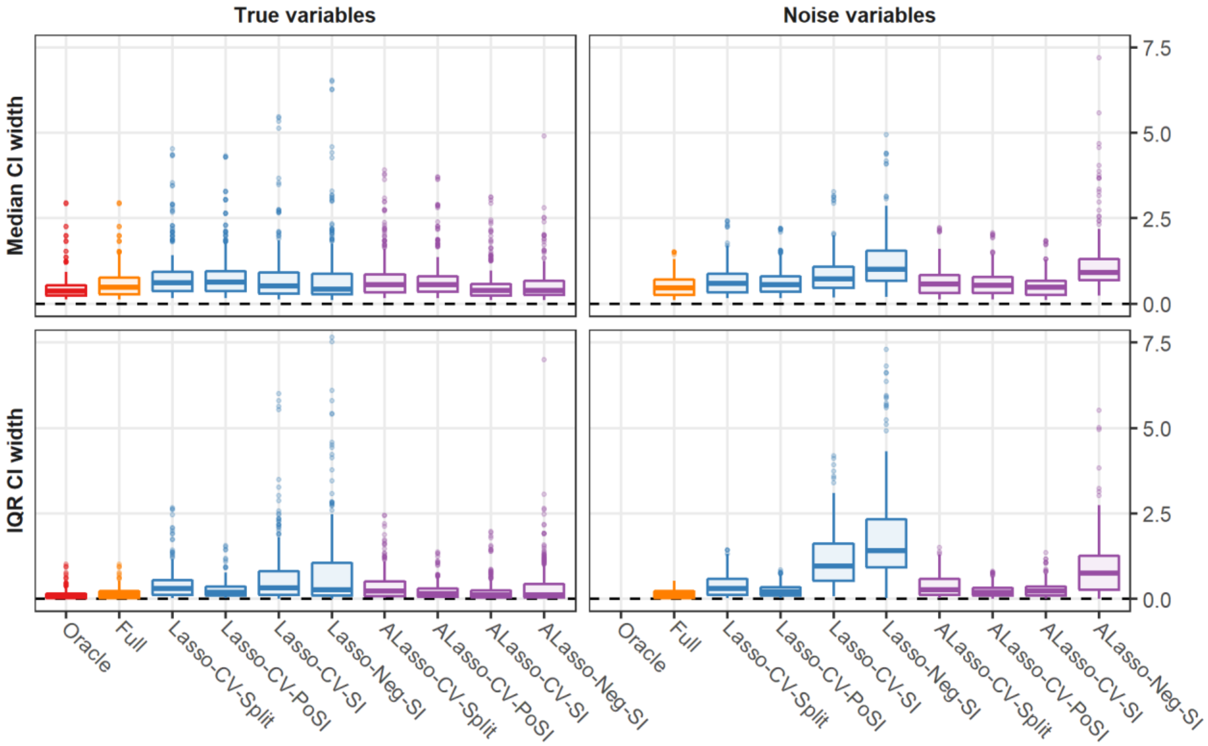

Supplementary Figure S 7: Toy simulation study: median and interquartile range (IQR) of width of selective 90% CIs for target R^2 of 0.8 stratified by true or noise predictor status. This figure provides a stratified view of Figure 3 in the main manuscript, for target simulation R^2 of 0.8 only. CIs were standardized. For each scenario the median and IQR of CI widths were computed, and over all variables and scenarios with specified target simulation R^2, the values were summarised by boxplots. In the left panel summarisation is done for true predictors, in the right panel for variables with true effect of zero (i.e. noise variables). Dashed lines mark a width of zero. Colors indicate the type of variable selection.

Results indicate that the widths for CIs are stable for most methods, but the SI approach shows increased median widths and variability for noise variables compared to true predictors.

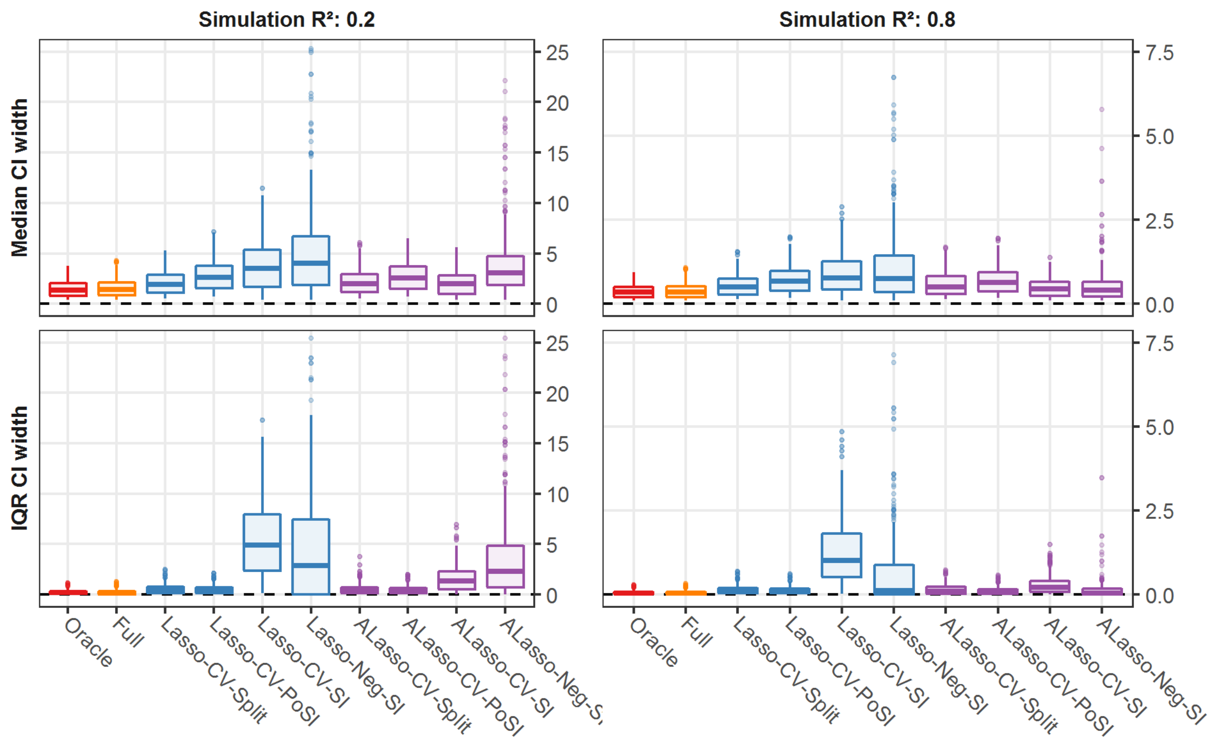

Supplementary Figure S 8: Realistic simulation study: median and interquartile range (IQR) of width of selective 90% CIs. CIs were standardized. For each scenario the median and IQR of CI widths were computed, and over all variables and scenarios with specified target simulation R^2, the values were summarised by boxplots. Dashed lines mark a width of zero.

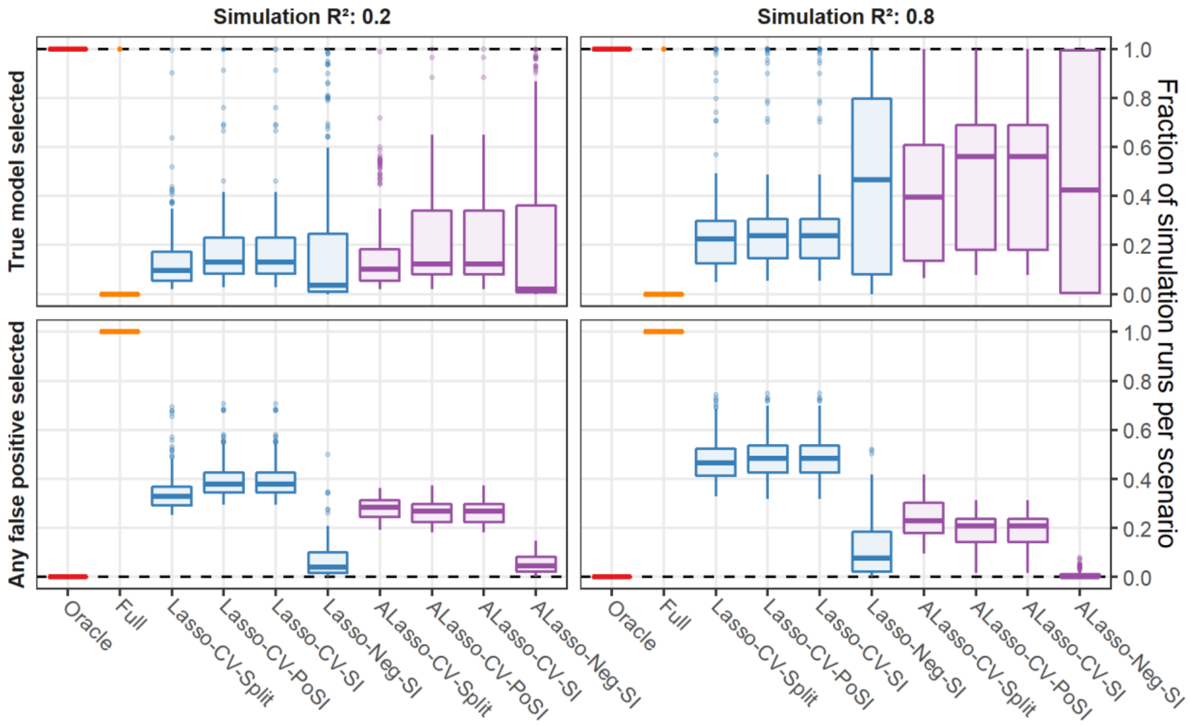

Supplementary Figure S 9: Toy simulation study: model selection. Only the two extreme R^2 values are depicted; for $R^2 = 0.5$ the results are in between. For each scenario, we computed the fraction of simulation runs in which the true data generating model (i.e. only true predictors) was selected (top row) and in which any false positive selection was made (i.e. a true non-predictor was selected, bottom row panel). The boxplots summarise these results over all scenarios. The target, optimal values are depicted as dashed lines. Colors indicate the type of variable selection.

Results indicate that the probability to select the true model increases with higher R^2 for the ALasso approaches, but remains largely constant for the Lasso approaches. Similarly, the probability to select false positives decreases for ALasso, but increases slightly for the Lasso.

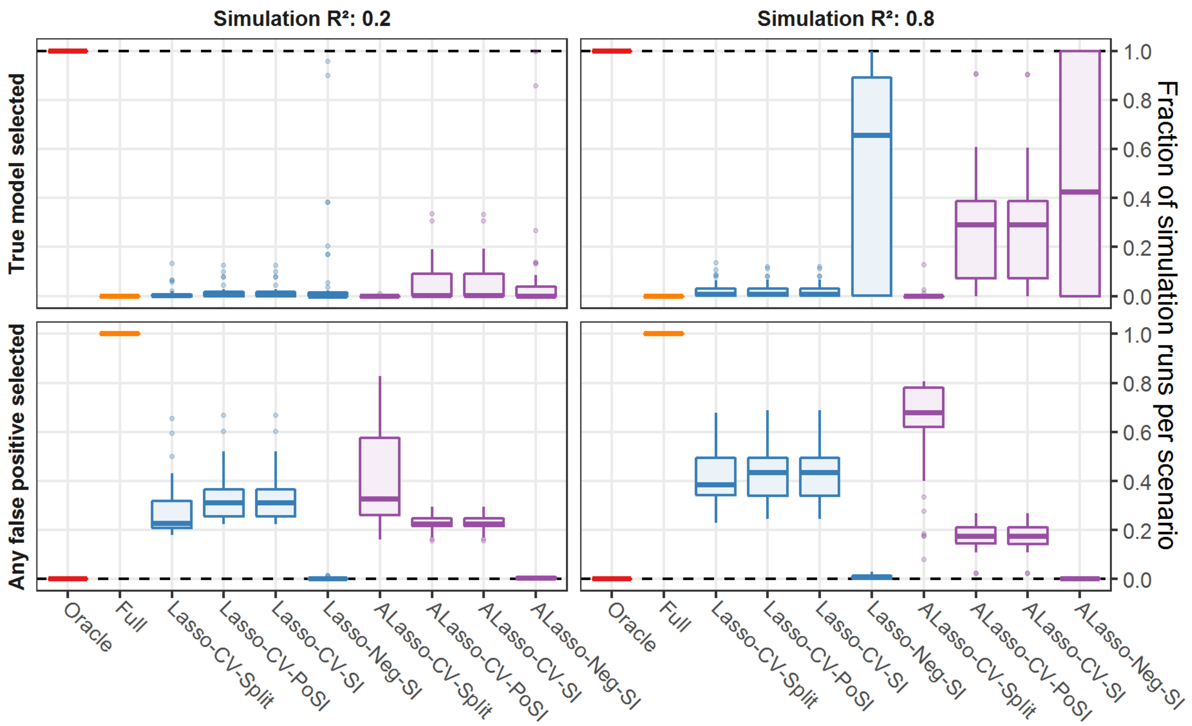

Supplementary Figure S 10: Realistic simulation study: model selection. Only the two extreme R^2 values are depicted; for $R^2 = 0.5$ the results are in between. For each scenario, we computed the fraction of simulation runs in which the true data generating model (i.e. only true predictors) was selected (top row) and in which any false positive selection was made (i.e. a true non-predictor was selected, bottom row panel). The boxplots summarise these results over all scenarios. The target, optimal values are depicted as dashed lines. Colors indicate the type of variable selection.

Results indicate that the probability to select the true model increases with higher R^2 for the ALasso approaches, but remains largely constant for the Lasso approaches. Similarly, the probability to select false positives decreases for ALasso, but increases slightly for the Lasso.

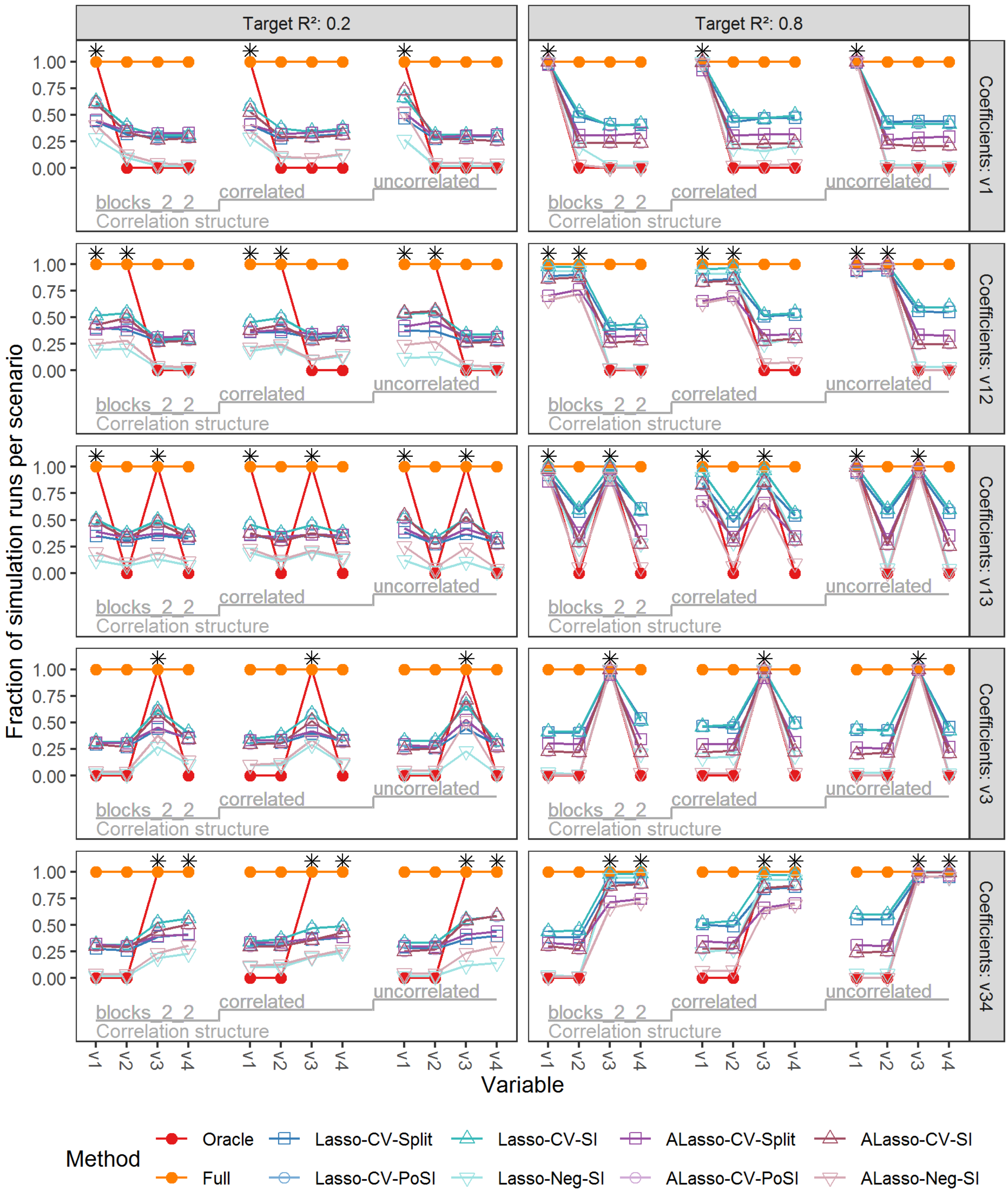

Supplementary Figure S 11: Toy simulation study: Nested loop plot of individual variable selection frequencies. Only a subset of all scenarios is shown: simulation R^2 of 0.5 and sample sizes 40 and 200 are excluded; only a few selected correlation structures (indicated by grey steps; see the Supplementary section 3 for details) and coefficient structures (rows,

Supplementary Table S 1) are depicted. Each dot represents the selection frequency for a specific variable over all simulation runs per scenario per method. Black stars indicate which variables have an effect. The red color is used for the Oracle model, which represents perfect selection and represents the full model view target for each variable selection procedure.

Similar plots were used to interpret the results from the simulation study. This example plot shows that Lasso-CV methods led to generally higher variable selection frequencies than ALasso-CV across the different simulation scenarios. The Lasso-Neg and ALasso-Neg approaches led to extremely sparse models. Furthermore, in case of correlation (as indicated by the "correlated" or "blocks_2_2" correlation structures) accuracy of selection was generally lower than in the uncorrelated case. Block-correlation also led to slightly different results than overall correlation for certain effect structure (e.g. "v34").

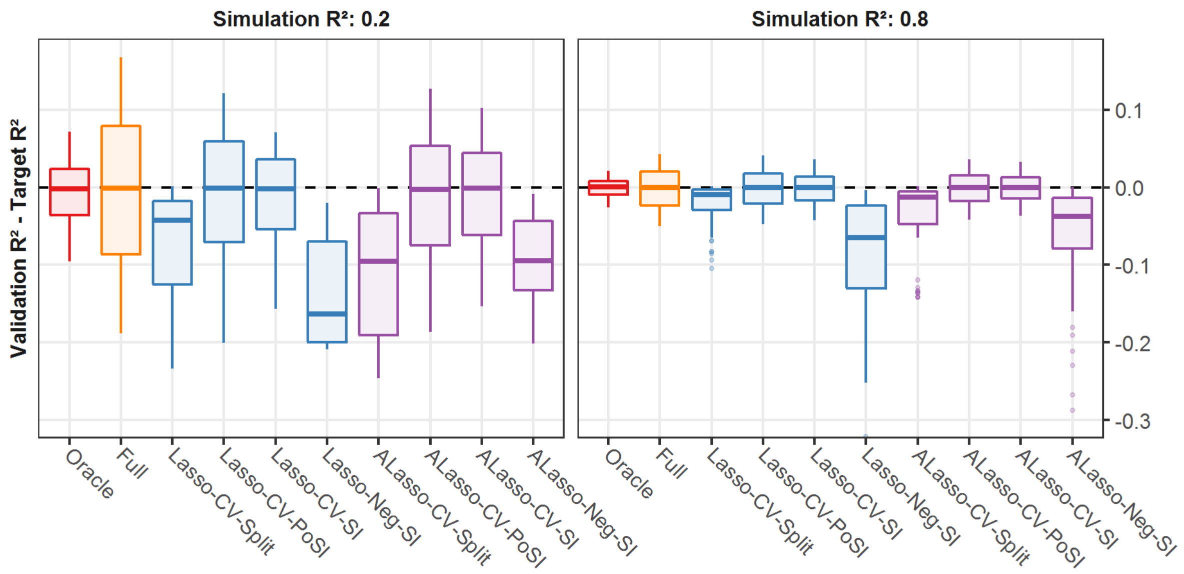

Supplementary Figure S 12: Realistic simulation study: predictive accuracy in terms of difference of validation R^2 and target simulation R^2. The target simulation R^2 was 0.2 in left panel, 0.8 in right panel. For each scenario, predictive accuracy was estimated by simulation, and over all scenarios, the values were summarised by boxplots. Dashed lines mark an optimal difference of zero. Colors indicate the type of variable selection.

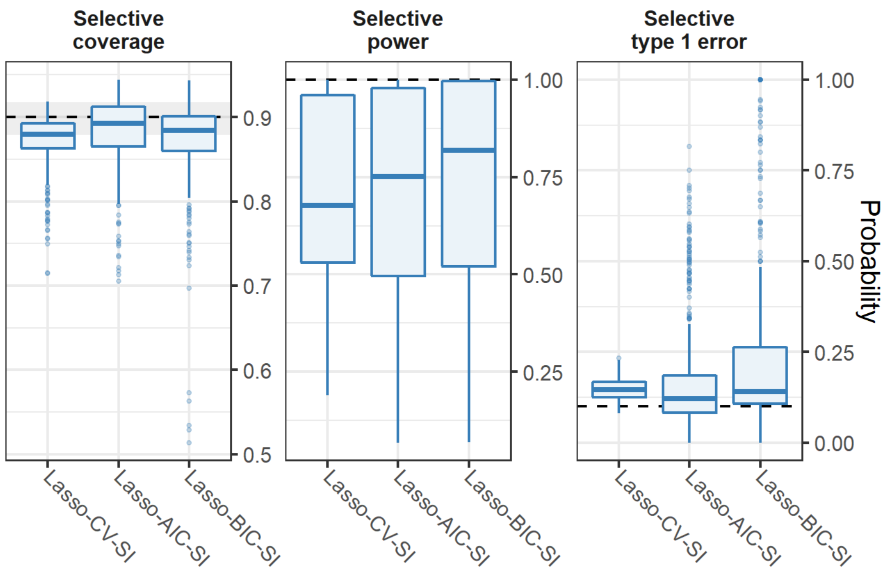

Supplementary Figure S 13: Extension of simulation study: primary estimands for Lasso using AIC and BIC based tuning for penalization strength instead of CV, and the SI method for selective inference. For comparison, the Lasso-CV-SI method from the main manuscript is included. For each scenario, the actual selective coverage rate, selective power and selective type 1 error were estimated by simulation, and over all scenarios, the values were summarised by boxplots. The target values are depicted as dashed lines (0.9 for coverage, 1 for power, 0.1 for type 1 error). Results for the primary estimands of all three methods are comparable in most scenarios. A closer comparison of how the different tuning methods affect the inference was out of scope for this manuscript.

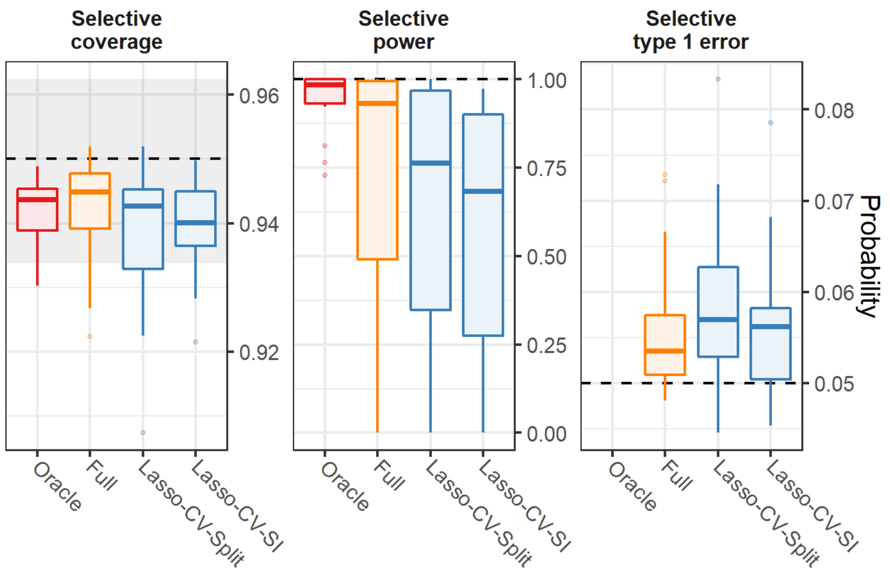

Supplementary Figure S 14: Extension of simulation study: primary estimands for Lasso logistic regression. This smaller study based on the realistic setup comprised scenarios in which the events per variable (5, 10, 50) and outcome prevalence (0.1 or 0.5) were varied. For each scenario, the actual selective coverage rate, selective power and selective type 1 error were estimated by simulation, and over all scenarios, the values were summarised by boxplots. The nominal confidence level was set to 0.95 in these simulations, and is depicted as dashed line. For selective power the dashed line indicates the target value of 1, for selective type 1 error the target value of 0.05. Colors indicate the type of variable selection. Monte Carlo error is indicated by grey areas describing binomial 95% CIs expected at the nominal confidence level with 900 iterations. Only four methods were evaluated in this smaller simulation setup, as the PoSI method is not directly available in this setting so far. Results are otherwise comparable to those of the realistic simulation setup in the main manuscript for linear regression.

4.1. Computational aspects

Computing time is an important aspect in practical applications, especially with many candidate predictors. As expected, the Lasso-CV-PoSI and ALasso-CV-PoSI approaches showed an exponential growth when comparing the toy and the realistic setup, while all of the other methods only showed minimal increases. The computation of the PoSI constant for a single iteration of the realistic setup with 17 candidate predictors took on average 35 seconds (Intel Core i7 4790 @ 3.6 GHz, R with multithreaded OpenBLAS matrix library). All the other methods remained well below 0.5 seconds.

References

1. Berk R, Brown L, Buja A, Zhang K, Zhao L. Valid post-selection inference. Ann Stat. 2013;41(2):802-37.
2. Negahban SN, Ravikumar P, Wainwright MJ, Yu B. A Unified Framework for High-Dimensional Analysis of M-Estimators with Decomposable Regularizers. Stat Sci. 2012;27(4):538-57.
3. Lee JD, Sun DL, Sun Y, Taylor JE. Exact post-selection inference, with application to the lasso. Ann Stat. 2016;44(3):907-27.
4. Friedman J, Hastie T, Tibshirani R. Regularization Paths for Generalized Linear Models via Coordinate Descent. J Stat Softw. 2010;33(1):1-22.
5. Tibshirani R, Tibshirani R, Taylor J, Loftus J, Reid S. selectiveInference: Tools for Post-Selection Inference. 2017.
6. Buja A, Zhang K. PoSI: Valid Post-Selection Inference for Linear LS Regression. 2017.
7. Kammer M. simdata: Generate Simulated Datasets 2020 [1.12.2021]. Available from: https://github.com/matherealize/simdata.
8. Binder H, Sauerbrei W, Royston P. Multivariable model-building with continuous covariates: 1. Performance measures and simulation. Germany: University of Freiburg; 2011.
